# 3RAD‐Guided SNP Discovery for Species Identification and Conservation of the Medicinal Southern African Tree Genus *Greyia* Hook. & Harv.

**DOI:** 10.1002/ece3.73412

**Published:** 2026-05-05

**Authors:** Iné Botha, Simo N. Maduna, Snorre B. Hagen, Namrita Lall, Dave K. Berger

**Affiliations:** ^1^ Department of Plant and Soil Sciences University of Pretoria Hatfield South Africa; ^2^ Forestry and Agricultural Biotechnology Institute (FABI) University of Pretoria Hatfield South Africa; ^3^ Department of Ecosystems in the Barents Region, Svanhovd Research Station Norwegian Institute of Bioeconomy Research (NIBIO) Svanvik Norway

**Keywords:** BioMark HD, genetic diversity, genotyping, medicinal plant, molecular systematics, phytomedicine, species identification

## Abstract

Accurate species identification is essential for conserving and managing plants that provide important ecosystem services and have ethnobotanical value. The *Greyia* tree genus (*G. sutherlandii*, *G. radlkoferi* and *G. flanaganii*) is endemic to South Africa and Eswatini, and certain genotypes have medicinal value for treating skin hyper‐pigmentation. However, distinguishing among species is difficult because of overlapping phenotypes and the limited resolution of standard DNA barcodes. To overcome these limitations, a robust molecular identification assay was developed using a two‐phase strategy. First, de novo SNP discovery using 3RAD sequencing identified 47,726 genome‐wide SNPs from two to three plants sampled from each species' core geographic range: *G. radlkoferi* in northern Limpopo, *G. sutherlandii* in eastern KwaZulu‐Natal, and *G. flanaganii* in the south‐eastern Eastern Cape. Principal component analysis and coancestry matrices revealed three discrete genetic clusters, supporting the recognition of the three species. Selecting a set of 200 SNPs with intermediate Fst values (0.2–0.5) resulted in optimal separation of the three clusters. This led to the final selection of a 23‐SNP panel that included five informative barcoding loci (ITS, *trnL‐F*, *matK*). Second, the 23 SNPs were converted into allele‐specific fluorescent PCR assays (SNP Type) for genotyping on the BioMark HD platform. The panel was validated using genomic DNA from 17 individuals from the 3RAD population groups and successfully differentiated all three species. It was then applied to 73 trees sampled across a 1000‐km transect from the Eastern Cape to Limpopo. Genetic clustering (PCA, UPGMA and ADMIXTURE) assigned each tree to one of three species‐level groups matching their expected ranges. In a practical case study, the assay also identified the species origin of 33 *Greyia* trees of unknown provenance from production orchards. This study provides an efficient SNP‐based tool for accurate species identification, supporting conservation planning and the sustainable management of *Greyia* populations.

## Graphical abstract

A two‐phase SNP discovery and assay‐development strategy was used to resolve species boundaries within the tree genus *Greyia*, as overlapping phenotypes and distributions limit accurate identification. From 47,726 genome‐wide SNPs generated by 3RAD sequencing, a diagnostic 23‐SNP Type panel was developed and validated, successfully distinguishing *G. radlkoferi* (green dots), *G. sutherlandii* (red dots), and *G. flanaganii* (blue dots) across their 1000‐km South African range. This assay reliably identified both wild and orchard‐grown trees, providing an efficient molecular tool for conservation and sustainable management of *Greyia* populations.

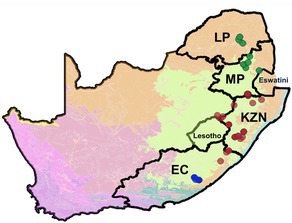

## 1 Introduction

An under‐studied group of plants of conservation genetics and medicinal importance is the tree genus *Greyia* Hook. & Harv. (order Geraniales) that is endemic to southern Africa (Dahlgren and Van Wyk 1988). Furthermore, genetic research on trees in Africa has lagged other parts of the world (Daru, Berger, et al. 2016). Three species are currently recognized: namely the Natal (or glossy) bottlebrush *Greyia sutherlandii* Hook. and Harv., the Wooly (or Transvaal) bottlebrush *Greyia radlkoferi* Szyszyl., and the Kei bottlebrush *Greyia flanaganii* Bolus (Botha et al. 2026). *G. radlkoferi* and *G. sutherlandii* are deciduous and predominantly found in the mist‐belt mountains of the Great Escarpment on the eastern side of South Africa mostly at altitudes greater than 1200 m (Killick and Kimpton 1977; Phillips and Gower 1923; Clark et al. 2011). *G. flanaganii* is an evergreen species of the Eastern Cape, occurring at lower altitudes on grassy hillsides and rocky steep slopes in restricted range along the Kei River and its tributaries (Steyn et al. 1999). These *Greyia* species perform valuable ecosystem services in the Grassland Biome of South Africa (Mbambezeli 2002, 2016; De la Cruz 2016). They have striking red inflorescences with copious nectar for generalist bird and insect pollinators (Botha et al. 2026).

*Greyia* species have medicinal value as bioprospecting efforts have revealed that leaf extracts of *G. radlkoferi* and *G. flanaganii* have anti‐tyrosinase activity (Mapunya et al. 2011; Lall et al. 2016). Tyrosinase is a multifunctional copper‐containing oxidase enzyme that initiates melanin synthesis in humans. The excessive accumulation of melanin pigments or the overexpression of tyrosinase can lead to skin disorders such as hyper‐pigmentation. Herbal formulations containing leaf extracts from these *Greyia* spp. are under development for commercialization. However, harvesting from the wild and plant poaching is a threat to the long‐term conservation of many ecosystems, especially if keystone species such as *Greyia* are targeted (Smith et al. 2023).

*Greyia* orchards have been planted in South Africa in attempts to provide sustainable sources for development of herbal remedies for treatment of skin hyper‐pigmentation (Malele et al. 2025). However, the origin and species identities of the *Greyia* orchard trees that were sourced from local nurseries is unknown, and morphological traits appear to be mixed (Ing et al. 2020). Due to the seasonal flowering and deciduous nature of *G. radlkoferi* and *G. sutherlandii*, field identification based on inflorescence traits or leaf morphology is challenging. Several authors have noted the unreliability of using leaf anatomy (e.g., wooly vs. glabrous leaves) to distinguish *G. radlkoferi* from *G. sutherlandii* (Steyn 1974; Dahlgren and Van Wyk 1988). Thus, there is a risk of adulteration of the herbal products with inferior ingredients, which highlights the need for an accurate species identification method.

Plant taxonomy has traditionally relied on morphological characteristics to classify and identify plant species (Dahlgren and Van Wyk 1988). Molecular systematics, which uses DNA sequence data to infer evolutionary relationships, is a complementary approach. This is based on comparison of phylogenetically informative DNA, such as chloroplast noncoding DNA or the internal transcribed spacer of the ribosomal DNA (ITS), that are mostly subject to neutral selection (Hollingsworth et al. 2011). In addition to defining species, molecular data has been used to prioritize conservation areas through “phylogenetic regionalization,” which, for example, has been applied to the southern African and Himalayan flora (Daru, Van der Bank, et al. 2016; Saqib et al. 2025).

Another important application of molecular data is DNA barcoding, which is used to catalog organisms within a study environment at species resolution (Hebert et al. 2003). Efficiency and cost‐effectiveness are achieved by a universal barcode, which in most animal lineages is provided by the mitochondrial *COI* gene (Hebert et al. 2003). In contrast, no single DNA barcode with comparable success has been identified for plants (CBOL Plant Working Group 2009). Most plant DNA barcodes are located in the chloroplast genome, although some nuclear loci have also been used as DNA barcodes, for example the rDNA ITS (Hollingsworth et al. 2011; Bolson et al. 2015). The two‐locus combination *matK* + *rbcL* plant barcoding approach endorsed by the CBOL Plant Working Group has a discriminatory efficiency of only 72%. Extending it to a multilocus method (including *atpF–atpH* spacer, *rpoB* gene, *rpoC1* gene, *psbK–psbI* spacer, and *trnH–psbA* spacer) did not clearly improve the species‐level discriminatory ability for plants (Li et al. 2015).

**FIGURE 1:**
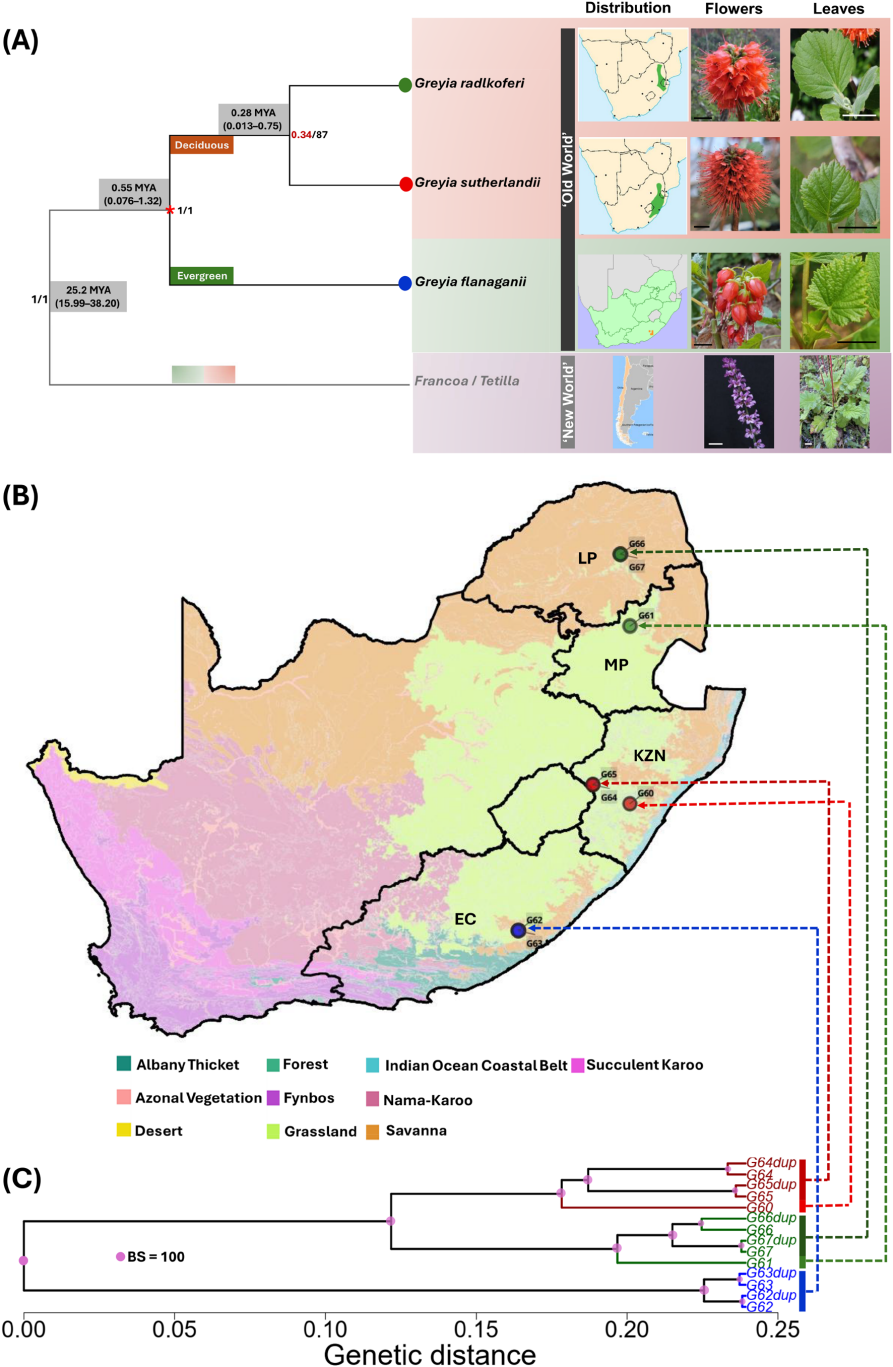
*Greyia* panel used for 3RAD‐guided species‐specific SNP discovery. (A) A representative topology of *Greyia*, the “Old World” clade, with the sister group *Francoa* + *Tetilla*, the “New World” clade, depicted as an outgroup, as adapted from Palazzesi et al. (2012) and Sytsma et al. (2014). The red asterisk indicates the well supported crown node of *Greyia*. The numbers adjacent to the nodes indicate the Bayesian posterior probability and maximum likelihood bootstrap values in the phylogenies of Palazzesi et al. (2012) and Sytsma et al. (2014). Shade boxes encompass divergence times derived from Sytsma et al. (2014). Species distribution, inflorescence and leaf morphology of *Greyia radlkoferi* , *Greyia sutherlandii*, *Greyia flanaganii* and *Francoa appendiculata* are shown on the right‐hand side. Scale bar = 2 cm. Image credits—*Greyia*: DK Berger; *Francoa*: M. Gardner, B. Greene. (B) Sampling sites of the *Greyia* ascertainment panel trees superimposed on the biome map of South Africa. Sample IDs refer to *G. radlkoferi* : P004_G66, P005_G67, P023_G61; *G. sutherlandii*: P090_G60, P242_G64, P245_G65; *G. flanaganii*: P160_G62, P161_G63. Provinces in South Africa: LP = Limpopo, MP: Mpumalanga, KZN = KwaZulu‐Natal, EC = Eastern Cape. (C) Genetic clustering of the *Greyia* ascertainment panel trees using 3RAD full SNP data set (*n* = 47,726). Samples and their duplicates (indicated by dup) are the same as listed in B and indicated with stippled lines. The clustering method used was UPGMA. BS refers to bootstrap values.

At the start of this project, the DNA barcode data for *Greyia* on GenBank was limited to five individuals of the three species, and the natural provenances of these plants were unknown. However, this *trnL‐F* and ITS barcode data was sufficient to differentiate *Greyia* from other related genera in the order Geraniales, and estimate divergence times for the three Greyia species (Palazzesi et al. 2012; Sytsma et al. 2014) (Figure 1A). More recently, phylogenetic analysis with *trnL‐F*, *matK, psbA‐trnH*, and ITS of five trees per “core population” of each *Greyia* species supported the recent divergence within the genus since the only significant barcode gap was for *psbA‐trnH*, which differentiated *G. flanaganii* from the other two species (Botha et al. 2026).

The advent of high‐throughput DNA sequencing technologies has led to the development of alternative DNA marker systems for species identification of plants (Yu et al. 2021). In recently diverged taxa (such as *Greyia*), these can be used to uncover phylogenetically useful polymorphisms by searching genome wide. They generally start with a SNP discovery step that can be achieved by different methods that include whole genome sequencing (WGS), genotyping‐by‐sequencing (GBS), or other reduced representation methods such as Restriction Site Associated DNA sequencing (RADseq) (Bayona‐Vasquez et al. 2019). In nonmodel or large genome plants, RADseq has the advantage of de novo SNP discovery. For example, this approach has been applied for plant species identification in *Camellia* L. (Yang et al. 2016), *Carex* section *Ceratocystis* Dumort (Nygaard et al. 2021), and in *Dendrolycopodium* A. Haines (Petlewski et al. 2022).

As discussed above, traditional plant barcoding using nuclear ITS or chloroplast loci relies on a few conserved sequences with moderate species resolution (CBOL Plant Working Group 2009), whereas SNP genotyping for genetic fingerprinting uses hundreds to thousands of genome‐wide markers, provides much higher discriminatory power for closely related taxa, but requires next‐generation sequencing and more complex analyses (Bayona‐Vasquez et al. 2019).

Applied population and evolutionary genetics are integral to the conservation and management of biodiversity, particularly in relation to plant genetic resources (Dempewolf et al. 2023). This can be viewed as the total hereditary material within an economically important plant species and its wild relatives, including all alleles of various genes (Salgotra and Chauhan 2023). Understanding the genetic diversity and biogeography of these populations is essential for their sustainable preservation, use and rewilding (Brown 1983; Salgotra and Chauhan 2023; Frisby et al. 2025). Conservation units (CUs) are defined as population units within a species to inform conservation efforts and management, as CUs determine conservation listing status (e.g., under state, provincial, or national jurisdictions) (Funk et al. 2012). A CU can be further divided into evolutionary significant units (ESU) which are populations within a species that have become sufficiently reproductively isolated to develop adaptations which makes them an evolutionary important sub‐component of the species (Funk et al. 2012). An example of ESUs would be ecotypes of *Arabidopsis thaliana* , which have developed tolerances to different pathogen diseases (Van der Linden et al. 2013). An ESU is defined by intraspecific genetic divergence, phenotypic, ecological, geographical and life history characteristics (Barbosa et al. 2018). The conceptual framework has been developed further by defining “evolutionarily sustaining conservation units” (ECSUs) (Hoelzel 2023). Importantly, these quantitative methods for conservation decision‐making are underpinned by genomics data, where neutral markers such as plant plastid genes and adaptive markers such as nuclear genes under selection can be used to define ECSUs in the biogeographical landscape.

This study had a main goal of developing a DNA‐based identification method to differentiate the three species of the medicinally important tree genus *Greyia*, considering the failure of standard DNA barcodes (Botha et al. 2026). A two‐step strategy was employed. First, triple‐enzyme restriction‐site associated DNA (3RAD) sequencing was employed for SNP discovery between a small set of carefully selected species‐representative *Greyia* trees. Second, a small panel of SNPs was selected for high throughput genotyping. This was applied to wild‐growing *Greyia* populations across their distribution range in South Africa as well as *Greyia* trees cultivated for their medicinal value. In addition, the SNP panel data was interrogated for patterns of distribution of *Greyia* across the country for future conservation planning.

## 2 Materials and Methods

### 2.1 Morphology and Sample Collection

The selection of wild‐growing *Greyia* trees for sampling was guided by records of the National Herbarium (PRE) of the South African National Biodiversity Institute (SANBI) located in Pretoria, the H.G.W.J. Schweickerdt Herbarium (PRU) at the University of Pretoria, South Africa, and GBIF (https://www. gbif.org/). In recent work, “core populations” of each *Greyia* species were defined and motivated based on proximity to type specimen provenances and morphologies that matched type descriptions (Botha et al. 2026). First, representative trees of each of the three species were selected from these core populations plus additional trees to broaden the genetic base (“ascertainment panel”) (Figure 1B). The ascertainment panel was made up of two trees from each “core population,” namely *G. radlkoferi* from near to Modjajiskloof, Limpopo Province, *G. sutherlandii* from Monks Cowl Nature Reserve, Central Drakensberg, KwaZulu‐Natal Province, and *G. flanaganii* from Nyara River, a tributary of the Kei River, Eastern Cape Province. An additional *G. radlkoferi* tree was included from the Verlorenkloof Estate population, Mpumalanga Province; this had the same morphology as the core population (wooly leaves and compact racemes made up of many open‐petalled [valvate] red flowers [> 30]) (Phillips and Gower 1923; figure 1A). The ascertainment panel also included an additional *G. sutherlandii* tree from Umgeni Valley Nature Reserve, KwaZulu‐Natal Province. This had the same morphology as the core *G. sutherlandii* population in the Central Drakensberg (glabrous leaves with elongated racemes of many valvate red flowers [> 30]) (Killick and Kimpton 1977; figure 1A). The *Greyia flanaganii* core population trees had glabrous leaves and the distinct pendulous urn‐shaped red flowers with unfused imbricate petals in racemes of less than 12 flowers (Steyn et al. 1999; figure 1A). Each *Greyia* tree was allocated a unique “P” number with additional G or RW codes that refer to a gDNA extraction sample. The ascertainment panel for de novo species‐diagnostic SNP discovery was therefore made up of three *G. radlkoferi* (P004_G66, P005_G67, P023_G61), three *G. sutherlandii* (P242_G64, P245_G65, P90_G60), and two *G. flanaganii* trees (P160_G62, P161_G63) (Figure 1B). The validation set was made up of 73 *Greyia* trees sampled across the distribution range from the Eastern Cape, KwaZulu‐Natal, Free State, Mpumalanga and Limpopo provinces (Appendix A: Table A1). In addition, 33 cultivated *Greyia* trees from orchards in Gauteng province were sampled (Appendix A: Table A1). Representative trees of each location were also sampled as herbarium voucher specimens and deposited at the HGWJ Schweickerdt Herbarium (PRU) at the University of Pretoria. Maps were generated using QGIS v. 3.34.15‐Prizren. The 2012 Vegetation Map obtained from SANBI was used as a biome map for South Africa (Rutherford et al. 2006).

### 2.2 DNA Extraction, 3RAD Library Preparation, and Sequencing

DNA was extracted from each ascertainment panel *Greyia* tree for 3RAD library preparation using fresh leaf material where available. This larger scale DNA extraction protocol, modified from Doyle and Doyle (1987) and Botha et al. (2026), is described in “DNA Extraction protocol 1 (for 3RAD library construction)” of Data S1.

In addition, a smaller scale method was used for DNA extraction from field sampled *Greyia* trees for SNP genotyping using an oKtopure DNA extraction robot with the sbeadex Maxi Plant DNA Purification Kit (LGC Genomics GmbH). This method is described in “*DNA Extraction protocol 2* (small scale for SNP genotyping from silica dried *Greyia* leaf material)” of Data S1.

The integrity and quantity of DNA extracted from the ascertainment panel plants was first evaluated on a 1% agarose gel, alongside a dilution series of lambda DNA (New England Biolabs, Massachusetts) ranging from 8 ng to 67 ng. Next, DNA quality and quantity was checked using the NanoDrop 2000 spectrophotometer (Thermo Fisher Scientific, Massachusetts) and the Quantus Fluorometer using the QuantiFluor ONE dsDNA System (Promega), respectively. Polymerase chain reaction (PCR) and restriction enzyme digest reactions were conducted to assess the purity of the DNA samples, to ensure that only trace amounts of inhibitory secondary metabolites copurified with the DNA and would have a negligible impact on downstream 3RAD library preparation steps and SNP genotyping on the Biomark HD (Data S1: Figure S1A–C). Each DNA sample was normalized to 10 ng/μL in nuclease and ion‐free water and working stocks were stored at −21°C prior to 3RAD library preparation.

RADseq libraries were prepared from the eight ascertainment panel *Greyia* trees separately and in duplicate using the Adapterama III library preparation protocol (Bayona‐Vasquez et al. 2019; their supplemental file SI), which is a modified version of double‐digest (dd)RAD (Peterson et al. 2012) that uses three enzymes for digesting genomic DNA (3RAD). This procedure reduces the amount of DNA required since the third enzyme inhibits the formation of adapter dimers and DNA chimeras during the simultaneous digestion and ligation reactions (Graham et al. 2015; Hoffberg et al. 2016; Bayona‐Vasquez et al. 2019). The literature was surveyed to identify frequently used enzymes in RADseq experiments of plants, and a pair of enzymes was chosen to be compatible with the respective 3RAD designs (Bayona‐Vasquez et al. 2019); their Table 1. The 3RAD enzyme combination *XbaI* (T|CTAGA) and *EcoRI‐HF* (G|AATTC) of equifrequent cutter enzymes with *NheI* (G|CTAGC) as the third enzyme for suppressing ligation of phosphorylated *NheI* adaptor dimers were selected. The EcoRI adaptor was not phosphorylated, which means that only a single strand of each *Greyia XbaI*—*EcoRI* fragment is used for library construction (Bayona‐Vasquez et al. 2019). A detailed account of the laboratory procedure of the 3RAD library preparation can be found in Data S1. Libraries were constructed for each *Greyia* tree as a technical replicate (duplicate) to estimate genotyping error rates. Cleaned and indexed library pools per design were sent to the Norwegian Sequencing Centre (NSC) for quality control and subsequent final size selection using a one‐sided bead clean‐up (0.7:1 ratio) to capture 550 bp +/−10% fragments, and the final paired‐end (PE) 150 bp sequencing on one lane of the Illumina NovaSeq 6000 platform.

**TABLE 1:** Novel SNP Type assays for species identification in *Greyia*.

SNP_NAME	Species^a^	ASP1_SEQ^b^	ASP2_SEQ^b^	LSP_SEQ^c^	STA_SEQ^d^
*Greyia*_3RAD_D1_16417.28	GFL	GCTAAACTCCCACCACATATGGA	GCTAAACTCCCACCACATATGGG	TCATGTCATCCTATTCCCTCATTTGCT	TCGAAAATACCCCTGGTCTAGATG
*Greyia*_3RAD_D1_16438.7	GFL	TTCACAATGCATGGAGTCTAGATGT	TTCACAATGCATGGAGTCTAGATGA	GGCATTGGCCATTATGTCATCCA	GTTAAGTCACACAGATGAAACAAAATGT
*Greyia*_3RAD_D1_16444.229	GRA	ACTGGGTATCTACTGGAAGCTATGAA	CTGGGTATCTACTGGAAGCTATGAG	CACTGGACGACTAATTGCATTTGTTCA	ACTGGGTATCTACTGGAAGCTATG
*Greyia*_3RAD_D1_16938.173	GRA	TGTCCTCAGTCTAGACTTGCAT	TGTCCTCAGTCTAGACTTGCAC	AGCGACAAGGCGTCGATGT	ACCCAGATTCCACCACCAAT
*Greyia*_3RAD_D1_16946.51	GSU	TACATAATTGAATGCATATTAAGTATTAATTAGACG	TACATAATTGAATGCATATTAAGTATTAATTAGACT	CCCTATGTGCAGAAGTGAGTATATAATGAAAGA	CGATGTTAAGATTATGTGCATGTTACATAATT
*Greyia*_3RAD_D1_17188.43	GSU	GAATACTCCATCTTTCACCTACTTTTTAATTCTG	GAATACTCCATCTTTCACCTACTTTTTAATTCTT	TCTTCTATGTTGTAGCATCGGAGATTGG	GTCCACACAAGTATCAAGTCAACT
*Greyia*_3RAD_D1_17357.154	GRA	AAATCTGAAACTAAAAATTTTCGTGAGTGAAAAC	CAAATCTGAAACTAAAAATTTTCGTGAGTGAAAAT	TGAAATTAATTTTTCGATTTTAATTTTCAGATTCAATTAA	ACTCGAAATAAAAAGCTACATCAAATCTGA
*Greyia*_3RAD_D1_17371.56	GSU	AAACCCTATACAAAAAAACACCAAACCTTA	ACCCTATACAAAAAAACACCAAACCTTG	AATTCATGCTGTTTTAGATTTTATACTGTTTGGTTGA	CAGCTATCCATTGAAACCCTATACAA
*Greyia*_3RAD_D1_17901.48	GSU	GAATAGACAATGGCCGCAAATGT	AGACAATGGCCGCAAATGC	CCCTCCTTCCTCCTCCTTTTCT	TGCAAATTATGAAGGCATTAGCAAGA
*Greyia*_3RAD_D1_18402.102	GSU	AATGAAGTTCATATGTTTTCACTATTCTGTACATTA	TGAAGTTCATATGTTTTCACTATTCTGTACATTG	AGATATAACATTAATTAGACATCTAAAATGAAAAAGTGAA	AAATGGTGAAGAGTCTTAATGAAGTTCATA
*Greyia*_3RAD_D1_19009.73	GFL	ACCCCTAGTCTTTATCACATTCAACTTC	CACCCCTAGTCTTTATCACATTCAACTTA	TCAATCTGTGCAAAAACAAAATTTTCAAAACCT	TTCGGTTTGCCTCAATTCTAAACA
*Greyia*_3RAD_D1_19082.71	GFL	AGAACACCAATGCTAGGTGTTCTC	AGAACACCAATGCTAGGTGTTCTT	CCAGTGATCTCCTTGCATCATCAAA	CCGTTAGCATTCAAGATACCCA
*Greyia*_3RAD_D1_19227.91	GFL	CGCCCTACGCGAAAATCAGT	GCCCTACGCGAAAATCAGG	CTCTCTACAAATTTTGTCGAAAAATTTCATGTAATGAGA	ACAGAGGAATCTCTCCGCC
*Greyia*_3RAD_D1_19287.52	GSU	ATTCTGGTTGGCATACTTTTTGCAT	TCTGGTTGGCATACTTTTTGCAC	CACACTCGAGCATATTGGCCAT	GGAAAGAGGCTCATCTTGTAATCA
*Greyia*_3RAD_D1_19922.176	GRA	CATTTATTTAAATTATTCGTTCACAATTATGAAACT	CATTTATTTAAATTATTCGTTCACAATTATGAAACA	TTTGTTTCAATATGATGATTATGTATTAAAGCTAATAGGC	CTGGTACATTTATTTAAATTATTCGTTCACAATTATG
*Greyia*_3RAD_D1_19983.195	GFL	CATAGTATGCATTGTTGTAATACCTGTATTTACG	CATAGTATGCATTGTTGTAATACCTGTATTTACA	GCATTTCTTTCTTGTTGGGGATGGA	GTGCTGATACCTCCTACTTTTGC
*Greyia*_3RAD_D1_20015.167	GRA	CCGGAAGCGACTTCTACC	CCGGAAGCGACTTCTACT	CGTAGCCGGCCGAGATCA	GAAGATGAGAAATTCCCTGCATTTT
*Greyia*_3RAD_D1_20179.46	GRA	TTAACCCTGTCCAATAAATGCATTATGG	GTTAACCCTGTCCAATAAATGCATTATGA	GTTAGCATATGCATGACATGATAACCCG	CTTGTTAACCCTGTCCAATAAATGC
*Greyia*_BarSNP_its2_01	GFL	GGGTAACTCCTCGAGCCTC	AGGGTAACTCCTCGAGCCTT	AGGACATACCTTCGCGGCA	CCGTGAACTCGTTACCTATCCC
*Greyia*_BarSNP_its2_02	GSU	GGGTGTCACGCACTCGTT	GGGTGTCACGCACTCGTC	TGCCCTACAATCCGATGACCA	GGGCACGTCTGCCTG
*Greyia*_BarSNP_its2_03	GFL	CCCTTCTGATCCTGTCGTGC	CCCTTCTGATCCTGTCGTGT	GGGTCTGTGAGCTCCTCGT	ACAAGCGGTGGTTGGTAAC
*Greyia*_BarSNP_matK_01	GFL	TCAAGAAGGACTCCAGAAGATGTTG	CTCAAGAAGGACTCCAGAAGATGTTA	CCTTTTTCTACGTAACCAATCTTCTCATTTACG	CCATAGAAATATATTCGCTCAAGAAGGA
*Greyia*_BarSNP_trnLH_01	GSU	CGCATCATCCTCATTTTACTAGATGC	ACGCATCATCCTCATTTTACTAGATGG	GGAATGATTCACAATCCATTTAATTGACATAGACC	CCCGACCATTTCCGACG

Abbreviations: GRA, *G. radlkoferi*; GSU, *G. sutherlandii*; GFL, *G. flanaganii*.

^a^ *Greyia* species with a unique nucleotide identified by each SNP assay compared to the other two species.

^b^ ASP refers to Allele Specific Primer. ASP1_SEQ and ASP2_SEQ differ only in the 3′ nucleotide, which is the SNP position. ASP1_SEQ corresponds to the sequence of the *Greyia* species identified by each SNP assay. These primers contain sequences complementary to the FAM and HEX probes, respectively, and are used together with the reverse Locus Specific Primer (LSP) for SNP genotyping. Primer sequences shown in 5′–3′ direction.

^c^ LSP refers to the reverse Locus Specific Primer.

^d^ STA refers to the forward Specific Target Amplification primer. The LSP and STA primers are used for pre‐amplification prior to SNP genotyping.

### 2.3 3RAD Data Processing and De Novo RAD Loci Assembly

After assessing the quality of the sequencing run using FastQC (Andrews 2010) and MultiQC v.2.31 (Ewels et al. 2016), the data was processed as follows. First, the raw sequences were demultiplexed, cleaned, and trimmed with the perl script *process_radtags.pl* included as part of Stacks v.2.51 (Rochette et al. 2019). The script was run using “inline_inline” mode because the internal barcodes are part of the sequence in both Illumina reads. Reads with an uncalled bases (−*c*) and low quality scores (−*q*) with a default sliding window of 15% of the length of the read and raw Phred score of 20, respectively, were discarded. We specified Read 1 and Read 2 restriction enzymes, and sequence tags (internal tags) and RAD‐tags (enzyme over‐hang) within 2 mismatches of their expected sequence (−*r*) were retained; otherwise, reads were discarded. The PE150 reads were truncated (−*t*) to 120 nt to have equal length among all reads with different barcodes.

Due to a lack of a *Greyia* reference genome or closely related species, the quality‐filtered reads were assembled into RAD loci using the de novo assembly pipeline with the perl script *denovo_map.pl* implemented in Stacks, which consisted of six components (*ustacks*, *cstacks*, *sstacks*, *tsv2bam*, *gstacks* and *populations*). To identify the parameters optimum for the de novo assembly of loci, Stacks was run on all samples to test different combinations of parameters including the *ustack* parameters *m* (the minimum depth of coverage required to create a stack) from 3 to 7, and *M* (the number of mismatches allowed between stacks to merge them into a putative locus) from 1 to 8; and the *cstack* parameter *n* (the number of mismatches allowed during the construction of the catalog) from *M* −1 to *M* +1 (Paris et al. 2017; Rochette and Catchen 2017). For each Stacks parameter (*m*, *M*, and *n*), the optimal value was determined following the approach outlined by Paris et al. (2017) and Rochette and Catchen (2017), referred to as the *r80* method. This method focuses on maximizing the utilization of biological information, thus generating a reliable set of loci, known as *r80* loci, essential for downstream analyses (Paris et al. 2017). A locus is deemed eligible for processing only if it is found in at least 80% of individuals within a specified (meta)population (Paris et al. 2017; Rochette and Catchen 2017). The effects of *M*, *m* and *n* values on summary statistics (i.e., average sample depth, number of SNPs retained, and number of loci retained) were visualized using RADstackshelpR (DeRaad 2021).

### 2.4 Species‐Diagnostic SNP Discovery, Filtering and Selection Workflow

#### 2.4.1 Step 1—SNP Calling

Due to the limited sample sizes for each species group of *Greyia*, Stacks was not rerun with a population map that assigned samples to the corresponding *Greyia* species groups. The global data outputted by the *populations* module for the optimum run were handled as a species complex, and downstream filtering was used to acquire a high‐quality panel of SNPs.

#### 2.4.2 Step 2—SNP Filtering

The package *SNPfiltR* (DeRaad 2022) was used to filter SNPs according to minor allele count (*min.mac* = 2), genotype depth (*depth* = 10), genotype quality (*gq* = 30) and data missingness (*cutoff* = 0.5). An arbitrary cutoff for missing data allowed per sample was initially set to investigate if missing data would influence clustering patterns between the species groups. SNPs were further filtered based on allele balance, the ratio of reads showing the reference allele to all reads, considering only reads from individuals called as heterozygous, and using the filter range 0.25–0.75 based on previous recommendations (Puritz et al. 2014; DeRaad 2022).

#### 2.4.3 Step 3—Genetic Diversity, Differentiation and Clustering Analyses

To characterize genetic diversity, observed heterozygosity (*H*_O_), gene diversity (*H*_s_), and *F*_ST_ per locus and overall loci were computed using the R package *hierfstat* v.0.5–11 (Goudet 2005; Goudet and Jombart 2022). Genome‐wide pairwise *F*_ST_ estimates were calculated using the Weir and Cockerham (1984) method with the function “*pairwise.WCfst*” in *hierfstat*, and computed the 95% CIs of *F*_ST_ values using the bootstrap function “*boot.ppfst*” (*nboot* = 1000) as implemented in *hierfstat*. Indices were considered significant if the 95% CI did not include zero. To investigate genetic clustering, a principal component analysis (PCA) using *SNPfiltR* was performed. Second, a matrix of Nei's genetic distance *D*_A_ (Nei 1972; Nei and Takezaki 1983) in StAMPP was computed for each pair of populations. The distance matrix was then used to create both a Neighbor‐Joining (NJ) and unweighted pair group method with arithmetic mean (UPGMA) dendrograms using the R package *ggplot2* v.3.3.5 (Wickham 2016) to describe broad patterns of genetic structure. Cluster support of the dendrograms was estimated using the “*aboot*” function in the R package *poppr* v.2.9.3 (Kamvar et al. 2014) with 1000 bootstrap replicates. Third, genomic admixture of *Greyia* was inferred using sparse nonnegative matrix factorisation (snmf) as implemented in the *snmf()* function in the R‐package LEA v. 3.8.0 (Frichot and Francois 2015; Gain and Francois 2021). Estimates of individual admixture coefficients over a range of *K* values (1–5) were calculated. The number of ancestral populations (*K*) was determined using the entropy criterion (Alexander and Lange 2011; Frichot and Francois 2015). Ancestry coefficients (*Q*) were visualized by plotting the *Q*‐values of each individual in a bar plot using the R package *pophelper* v.2.3.1 (Francis 2017). Last, the genomic coancestry among individuals was assessed with fineRADstructure (Malinsky et al. 2018). To do this, a coancestry matrix was inferred with the script *RADpainter*. Subsequently, clustering was done with the Markov chain Monte Carlo method of fineRADstructure, running for 500,000 generations and sampling every 1000 generations; the first 200,000 generations were discarded as burn‐in (nondefault parameters: *x* 200,000 *y* 300,000 *z* 1000). A tree for visualization was inferred with fineRADstructure using the tree‐building algorithm of Lawson et al. (2012) with 10,000 attempts (nondefault parameters: *m*T *x* 10,000). fineRADstructure results were plotted with R scripts included in the fineRADstructure package.

#### 2.4.4 Step 4—Final SNP Selection for SNP Type Assay Panel Development

Subsets of 200 SNPs were selected based on per locus *F*_ST_ values as follows: *Data Subset 1* included SNPs with *F*_ST_ values in the range 0.3 to 0.5; Data Subset 2 *F*_ST_ = 0.5–0.7; and Data Subset 3 of SNPs with *F*_ST_ = 0.8–1. PCA was performed to identify the subset with PC1 and PC2 explaining highest variation with three distinct clusters and reconstructed the fineRADstructure coancestry matrix to validate the genetic clustering of samples. The initial goal was to design a Flex Six SNP Type chip, which would include a final set of 12 species‐diagnostic SNP assays for rapid, reliable, and cost‐effective large‐scale SNP genotyping of *Greyia* trees. Therefore, from the identified “species‐diagnostic” subsets of 200 SNPs a total of 19 SNPs were selected for SNP Type assay design and characterization. Furthermore, to comply with Standard Biotools' stipulation of a minimum purchase of 24 SNP Type Assays (Extra Small), we incorporated five SNPs found in plant DNA barcodes (three variants from *ITS*, one variant from *trnLF*, and one from *matK*) previously characterized in *Greyia* species (Botha et al. 2026) for further characterization with the larger sets of wild and cultivated *Greyia* trees.

### 2.5 SNP Type Assay Design and Panel Optimization

Multiple sequence alignments of alleles from all ascertainment panel samples for each RAD loci were constructed and the position of SNPs was annotated. Any regions where any two SNPs were within 30 bp were excluded from assay design to avoid SNPs in primer binding sites. SNP Type assays for the 24 SNPs were designed with the web‐based Standard Biotools D3 assay design tool (https://d3.standardbio.com) under default settings. One SNP failed and Standard Biotools delivered 23 SNP Type assays, where each assay consisted of three types of primers: one specific target amplification (STA) primer, two allele‐specific primers (ASPs) and one reverse locus‐specific primer (LSP). When either the quantity and/or the quality of the input DNA is not ideal, a Specific Target Amplification (STA) is initially performed to enrich the amplicon including the targeted SNP sequences using the STA and LSP primer pair (Wang et al. 2009; Bhat et al. 2012). The ASPs and LSP are used for SNP genotyping, with ASP1 and ASP2 containing complementary sequences to the FAM and HEX probes, respectively, enabling differentiation between the two SNP alleles (diploid organisms, three possible genotypes).

The SNP Type assays were initially tested by genotyping six ascertainment panel plants (2 trees per *Greyia* species) previously used for 3RAD‐guided SNP discovery using the Flex Six SNP Type Genotyping Kit and run on the Biomark HD (Standard BioTools Inc., formerly Fluidigm) following the manufacturer's instructions. The Flex Six Genotyping integrated fluidic circuit (IFC) consists of six 12‐assay‐by‐12‐sample partitions that can each be run separately or together (Standard Biotools). Because PCR inhibitory secondary metabolites can copurify with plant DNA, we subsequently tested whether undiluted and 1:10 diluted DNA would yield different call rates, genotype clustering profiles, and error rates. Last, we tested STA on undiluted and 1:10 diluted DNA samples, the number of PCR cycles for STA, and the post‐STA dilution ratio of the amplicons. We slightly modified the manufacturers' original STA protocol during optimization. STA‐PCRs contained 1.25 μL of DNA, 2.5 μL 2× QIAGEN Multiplex PCR Master Mix (Qiagen), and 0.5 μL of 10× SNP Type Assay STA primer pool (500 nM). Thermal conditions included an initial denaturation step of 95°C for 15 min, followed by 12–20 cycles of 95°C for 15 s and 60°C for 4 min. STA products were diluted 1:10, 1:20, and 1:100 with low salt TE Buffer. Next, fluorescently labeled allele‐specific primers were used to target both alleles in a genotyping PCR according to the manufacturer's protocol. All samples were run as duplicates along with one no template control (NTCs) reaction and one no assay control (NAC) per partition of the IFC to monitor for potential contamination and background fluorescence, respectively.

The SNPs were scored using the Fluidigm SNP Genotyping Analysis Software v. 4.5.1 and manually validated the automatically generated scatter plots. Sample replicates with a call rate of ≤ 85% across all SNP loci were excluded from the clustering algorithm. Furthermore, we excluded NTCs exhibiting significant fluorescence (≥ 0.2) to ensure accurate normalization of fluorescence data across all loci. The occasional fluorescence of NTCs in the Biomark HD system is a recognized phenomenon; however, it does not warrant significant concern, as target DNA is preferentially amplified in genotyping PCRs containing sample DNA (Kraus et al. 2015; Standard BioTools). We compared the SNP calls for each sample with those obtained from 3RAD for each control ascertainment panel tree. Subsequently, the 23 SNP Type assays were evaluated on a larger panel of 17 Greyia trees that included the ascertainment panel samples and additional trees from the same locations.

### 2.6 Validation of SNP Markers and Scoring Procedure

To evaluate the performance of the 23 SNP Type assays for standardized genetic species identification, the SNPs were characterized using a diverse panel of 73 *Greyia* trees sampled across the distribution of *Greyia*. The 3RAD ascertainment panel was included as genotype reference controls. *Melianthus major* L., *Melianthus minor* L., *Melianthus comosus* Vahl, and *Bersama lucens* (Hochst.) Szyszyl. growing at the Manie van der Schijff Botanical Gardens, University of Pretoria were included as outgroups for assay specificity testing. For the purposes of efficiency, the samples were analyzed using two 96.96 Dynamic Array IFC so that each assay could be run for each sample in quadruplicates (23 assays plus one NAC) per IFC for validation of genotypes and error estimation. The optimized genotyping protocol involved 1:10 DNA dilution followed by two consecutive PCR reactions. In the first one, all 23 loci were preamplified in a single reaction with locus‐specific primers (STA reaction) using 14 cycles in order to dilute any inhibitory secondary metabolites that may be present in DNA samples. Next, a genotyping PCR was performed according to the manufacturer's protocol on the Biomark HD and SNP calling was done using the Fluidigm SNP Genotyping Analysis Software following the procedure outlined above.

To mitigate the impact of locus‐specific performance on the evaluation of replicate genotypes, the success rate of each locus was assessed, and those that amplified in less than 80% of the reactions were removed. Sample performance was assessed by determining the SNP call rate for each sample, defined as the proportion of successfully called genotypes or amplifications across all loci for that sample. A mean call rate for each sample was computed as the average of the call rates, given that each sample was genotyped four times. Genotype consistency among replicates was evaluated by tallying the number of loci that exhibited agreement or disagreement in genotype, or alternatively, had missing data across the four PCR duplicates of a sample at each locus. If a minimum of two PCR replicates contained no genotype data, the locus was classified as having insufficient or missing data. To establish consensus genotypes, the most prevalent allele was presumed to be correct according to the inferred consensus‐building criteria—for example, if three replicates exhibited consistent homozygous genotypes while a fourth replication had a heterozygous genotype. If two of the replicates were heterozygous and the other two exhibited contrasting homozygous genotypes, the genotype was inferred to be heterozygous. The percentage of alleles matching (Match Score) between the four consensus genotypes attributed to the same individual identity was computed using Microsatellite Toolkit (Park 2001). The genotyping error rates were defined as 100 minus the Match Score.

One replicate with the highest call rate was retained and GenAlEx v. 6.503 (Peakall and Smouse 2006, 2012) was used to perform a multilocus matches analysis for characterizing the number of unique and repeated multilocus genotypes (MLGs). Descriptive genetic diversity statistics per locus, namely observed heterozygosity (*H*_O_), unbiased gene diversity (*H*_S_), and polymorphic information content (*PIC*) were calculated in Microsatellite Toolkit. The allele sharing distance of Bowcock et al. (1994) as implemented in Microsatellite Toolkit was calculated and the output allele sharing matrix (ASM) was assessed as a putative approach for assigning unknown samples to the three species of *Greyia*. To determine species identity for unknown samples, a minimum threshold of 80% shared genotypic information with control plants was employed. Additionally, the allele sharing distance was used to reconstruct a dendrogram with the UPGMA, average linkage clustering method (Nei 1975), using the Neighbor program from the PHYLIP package v. 3.698 (Felsenstein 1993).

A principal coordinates analysis (PCoA) was perfomed in GenAlEx to assess the patterns of genetic clustering of control trees and the unknown wild and cultivated *Greyia* trees. Additionally, the number of discrete genetic clusters (*K* or *k*) and individual proportional population assignment to each cluster (membership or ancestry coefficient, *Q* or *q*) were inferred using two approaches: (1) Discriminant Analysis of Principal Components (DAPC), a multivariate approach that does not require population‐genetic models such as Hardy–Weinberg and/or linkage equilibrium (Jombart et al. 2010); and (2) ADMIXTURE, a model‐based maximum likelihood clustering approach (Shringarpure et al. 2016). The R package *adegenet* v.2.1.1 (Jombart 2008) was used to perform DAPC on clusters predefined by species for control samples and unknown samples using the *dapc()* function. For DAPCs without prior group information, the number of discrete genetic clusters was inferred using the *find*.*clusters()* function in *adegenet*, which runs successive *K*‐means clustering with an increasing number of clusters (*k*). Ten independent runs of the *find.clusters* function were conducted with the *diffNgroup* option selected to identify the sharp changes in fit of models with different number of clusters based on the Bayesian information criterion (BIC) score. Then, the optimal *k* was selected by applying the BIC score as recommended by Jombart et al. (2010). For all DAPC analyses, the number of principal components to retain was determined using the cross‐validation approach implemented by the function *xvalDapc()* with 100 repetition in *adegenet*. ADMIXTURE was run as implemented within the AdmixPipe v.3 pipeline of (Mussmann et al. 2020). To determine the best‐fit number of clusters (*K*) for the data, *K* values from 1 to 6 were assessed with 20% cross‐validation (CV). Twenty replicates of ADMIXTURE were run at each *K*, and the best‐fit *K* was determined as the value that had the lowest average CV score across replicates. Additionally, given that the sampling of putative *Greyia* species was uneven, the Puechmaille (2016) method was applied to determine the optimum *K* as implemented on the STRUCTURESELECTOR web server (http://lmme.qdio.ac.cn/StructureSelector/; Li and Liu 2018). The ADMIXTURE results were visualized using the CLUMPAK server (http://clumpak.tau.ac.il/; Kopelman et al. 2015). In both approaches, the levels of admixture i.e., fractions of individual genomes that belong to different ancestry groups, were determined by using a cut‐off value of *q* < 0.8.

*Greyia* trees were assigned to species genetic clusters based on the genetic clustering analysis and estimated genetic diversity descriptors and pairwise *F*_ST_ used GenAlEx. Furthermore, to evaluate the accuracy of this species assignment approach, Nei's genetic identity (*I*) within and between species genetic clusters was calculated and population assignment tests were performed in GenAlEx.

## 3 Results and Discussion

### 3.1 Strategy for SNP Discovery and Assay Design

**FIGURE A1:**
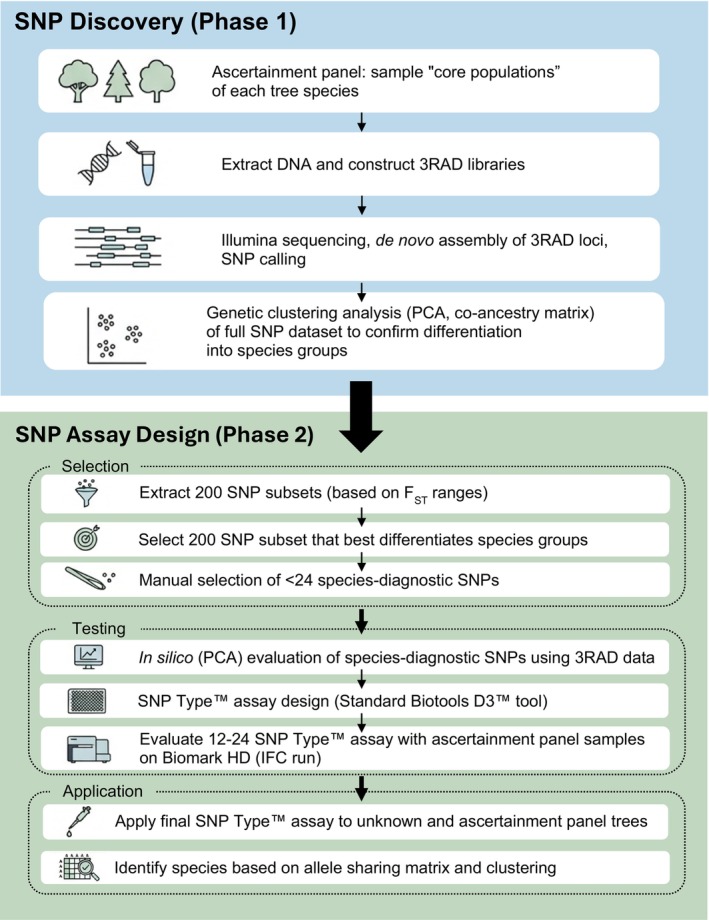
Two‐phase strategy for the development of an SNP‐based species identification method for the genus *Greyia*. The ascertainment panel was made up of 2–3 trees of each species *G. radlkoferi*, *G. sutherlandii*, and *G. flanaganii* sampled from geographically separated “core populations.” The SNP assay design started with extracting 200 SNP subsets from the full SNP dataset based on F_ST_ ranges (0.3–0.5, 05–0.7, 0.8–1). In this study, the subset in the 0.3–0.5 range was selected based on PCA and co‐ancestory analyses. An optional step was including informative SNPs from standard barcoding genes in the final SNP assay design. Proof of concept evaluation (testing) of the 12–24 SNP panel is carried out on the Flex Six Genotyping integrated fluidic circuit (IFC) run on the Biomark HD instrument. Screening of large numbers of unknown samples on the Biomark HD instrument was performed on the 96.96 Dynamic Array IFC to obtain technical replicates of assays/samples in a single run. Final species identifications made with the allele scoring matrix were confirmed with other genetic clustering methods (e.g., PCoA, Admixture, and DAPC). This framework of SNP discovery and SNP assay design can be adapted to develop a 12–24 SNP panel for species identification/genotype differentiation in other plants/organisms.

The two‐phase strategy for SNP discovery and SNP assay design for *Greyia* species identification is illustrated in Appendix A: Figure A1. This entailed de novo SNP discovery using 3RAD of species‐representative individuals followed by the design of a SNP assay for genotyping.

### 3.2 3RAD Datasets and Species‐Diagnostics SNPs

A total of 405,492,844 raw paired‐end reads were generated after Illumina sequencing of 3RAD libraries from the ascertainment panel of the three *Greyia radlkoferi* , three *Greyia sutherlandii*, and two *Greyia flanaganii* species‐representative trees (Figure 1B). The processing of this raw data with the program *process_radtags* resulted in the removal of 4.9% of the data due to poor sequencing quality, adapter contamination, ambiguity in the restriction site and ambiguous barcodes (inability to attribute a sequencing read to an individual). Overall, a high percentage of retained reads (95.1%) with a mean of 16.1 million reads per sample were recovered. After removing samples that did not sequence well, the de novo assembly procedure in Stacks generated a catalog of 370,059 putative RAD loci with a mean effective per‐sample coverage of 101.6× (min = 18.4×, max = 227.2×). Following the methods described in section 2.3, the optimum Stacks parameter values for assembly of these RAD loci were *m* = 3, *M* = 7 and *n* = 8 (Data S1: Figure S2). After SNP filtering, a final panel of 47,726 polymorphic SNPs (minimum coverage = 10×; genotyping rate = 0.82; *n* = 14) was retained. The SNP genotypes between technical replicates were largely consistent with a mean error rate of 2.6% (min = 0.47%, max = 9.8%).

**FIGURE A2:**
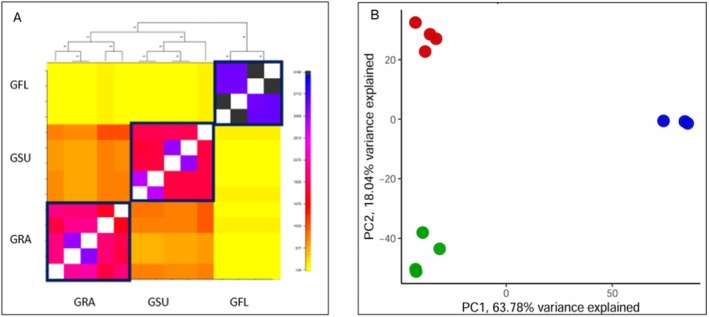
Genetic clustering of the eight *Greyia* trees (ascertainment panel) using the 3RAD full SNP dataset. (A) The genetic clustering was evaluated using a co‐ancestry matrix using the full SNP (*n* = 47,726) dataset derived from 3RAD libraries from eight *Greyia* trees (including replicate libraries). The level of genetic similarity is indicated by the color scale to the right. The darker color indicates a higher level of genetic similarity. Genetic clusters are indicated by blue squares forming three distinct blocks. The *G. radlkoferi* (GRA) samples included P023_G61, P004_G66, P004_G66_dup, P005_67, P005_67_dup. The *G. sutherlandii* (GSU) samples included P090_G60, P242_G64, P242_G64_dup, P245_G65, P245_G65_dup. The *G. flanaganii* (GFL) samples included P160_G62, P160_G62_dup, P161_G63, P161_G63_dup. The white blocks are samples as compared to themselves, and thus, the data are not considered. (B) The clustering was also represented by PCA of the same samples and SNP dataset. PC1 (68.78% variance explained) differentiates GFL from GSU/GRA, whereas PC2 (18.02% variance explained) differentiates all three species groups. Red dots = GSU, green dots = GRA, blue dots = GFL.

Genetic clustering with the 47,726 SNP dataset was explored using several independent methods, and genetic structuring of the eight ascertainment‐panel *Greyia* samples (including technical replicates for six of them) matched the three recognized species (Figure 1C, Appendix A: Figure A2). There was no evidence to suggest that the relatively higher error rate among technical replicates affected genetic grouping. The postprocessing of the *snmf* results based on the lowest average CV score across replicates identified *K* = 3 as the most likely number of genetic clusters for the ascertainment‐panel trees for de novo species‐diagnostic SNP discovery. This was consistent with the number of clusters based on the first two principal components of the PCA analysis (PC1 and PC2 accounting for 86.8% of cumulative variation) and fineRADstructure coancestry matrix and dendrogram (Appendix A: Figure A2).

**FIGURE A3:**
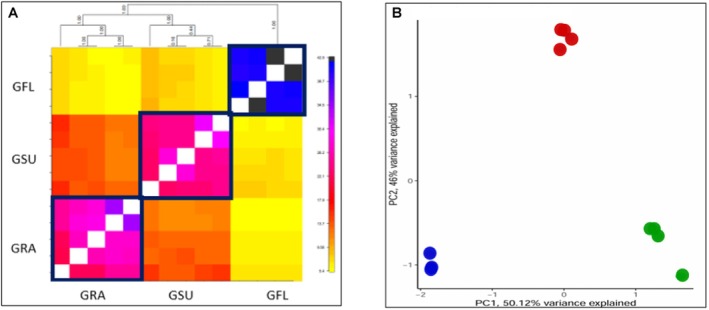
Genetic clustering of the eight *Greyia* trees (ascertainment panel) using the 200 SNPs selected from the 3RAD dataset. (A) The genetic clustering was evaluated using a co‐ancestry matrix using the 200 SNP subset (*n* = 200, *F*_*ST*_ = 0.3–0.5) derived from 3RAD libraries from eight *Greyia* trees (including replicate libraries). The level of genetic similarity is indicated by the color scale to the right. The darker color indicates a higher level of genetic similarity. Genetic clusters are indicated by blue squares forming three distinct blocks. The *G. radlkoferi* (GRA) samples included P004_G66, P004_G66_dup, P005_67, P005_67_dup, P023_G61. The *G. sutherlandii* (GSU) samples included P242_G64, P242_G64_dup, P245_G65, P245_G65_dup, P090_G60,. The *G. flanaganii* (GFL) samples included P160_G62, P160_G62_dup, P161_G63, P161_G63_dup. The white blocks are samples as compared to themselves, and thus, the data were not considered. (B) The clustering was also represented by PCA of the same samples and SNP dataset. PC1 (50.12% variance explained) differentiates all three species groups, whereas PC2 (46% variance explained) differentiates GSU from GFL/GRA. Red dots = GSU, green dots = GRA, blue dots = GFL.

**FIGURE A4:**
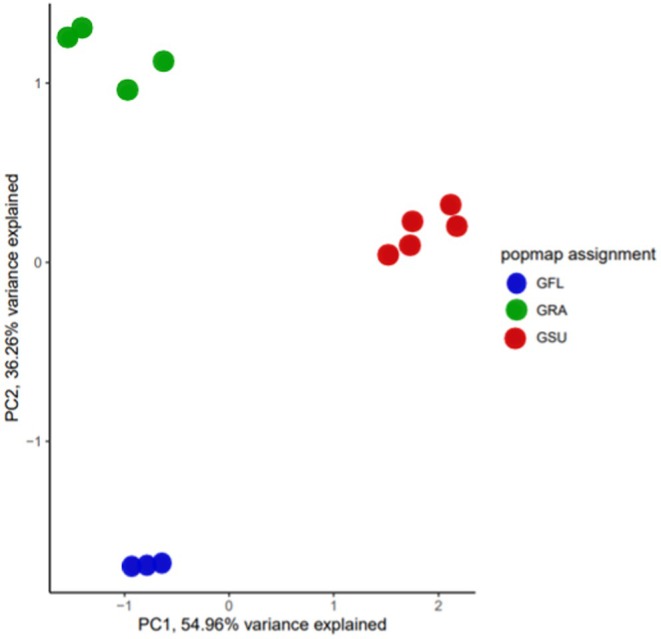
PCA of the 3RAD data for the 19 selected nuclear SNPs for the three *Greyia* species (ascertainment panel). Dots represent individual samples (*n* = 14). Sample IDs refer to the following Greyia trees, including duplicates: *G. sutherlandii* (GRU): P242_G64, P242_G64_dup, P245_G65, P245_G65_dup; P090_G60; *G. radlkoferi* (GRA): P004_G66, P004_G66_dup, P005_G67, P005_G67_dup, P023_G61; *G. flanaganii* (GFL): P160_G62, P160_G62_dup, P161_G63, P161_G63_dup. Red dots = GSU, green dots = GRA, blue dots = GFL. PC1 explains 54.96% of the variability while PC2 explains 36.26% of the variability and differentiates the three species groups.

Upon evaluating three distinct *Greyia* data subsets, each comprising 200 SNPs with varying *F*_ST_ values, it was determined that Data Subset 1 (*F*_ST_ = 0.3–0.5) revealed the most similar genetic clustering pattern to the full SNP dataset for the eight tree *Greyia* ascertainment panel. Both fineRADstructure and PCA analyses demonstrated closely‐knit species genetic clusters, with PC1 and PC2 accounting for a cumulative variation of 96.12% (Appendix A: Figure A3). From Data Subset 1 of 200 SNPs, a small panel of 19 species‐diagnostic SNPs that recovered the same genetic clustering patterns as both the full and subset 3RAD datasets was identified, with PC1 and PC2 accounting for a cumulative variation of 91.22% (Appendix A: Figure A4).

### 3.3 *Greyia* 23 SNP Type Assay Design and Validation

**FIGURE A5:**
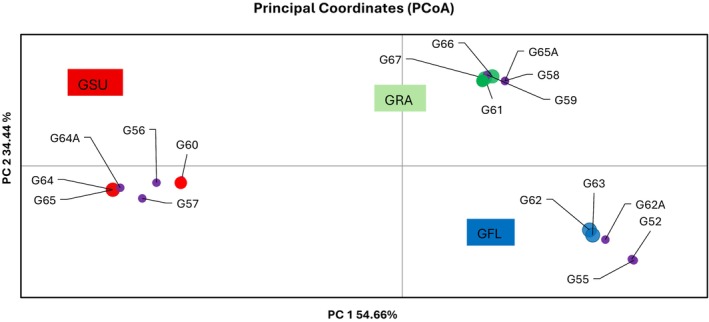
*Greyia* 23 SNP Type^TM^ assays differentiate the ascertainment panel trees into three species groups. Principal coordinates analysis (PCoA) with the 23 SNP data from the Biomark HD microfluidics system is shown for the Greyia ascertainment panel trees with three additional trees from each of the core geographic sites in Limpopo, KwaZulu‐Natal, and Eastern Cape representing *G. radlkoferi* (GRA), *G. sutherlandii* (GSU) and *G. flanaganii* (GFL), respectively (n=17). Sample IDs refer to the following Greyia trees with ascertainment panel members in bold: *G. sutherlandii*—**P090_G60**, P241_G64A, **P242_G64**, P243_G56, P244_G57, **P245_G65;** *G. radlkoferi*—P001_G58, P002_G59, P003_G65A, **P004_G66, P005_G67, P023_G61;** *G. flanaganii*—P157_G52, P158_G55, P159_G62A, **P160_G62, P161_G63**. Red dots = GSU, green dots = GRA, blue dots = GFL, purple dots = additional trees. PC1 explains 54.66% of the variability, while PC2 explains 34.44% of the variability.

The 23 SNP Type assays on the Biomark HD microfluidics system were successfully designed and optimized for standardized genetic species identification of *Greyia* trees (Table 1). This included 18 SNPs from the 3RAD data and five SNPs from *ITS*, *trnLF* and *matK* barcodes (from Botha et al. 2026). As proof of concept, it was first confirmed that the *Greyia* 23 SNP Type assay could recover consistent multilocus genotypes (MLGs) that differentiated between the three species using 17 *Greyia* trees made up of the ascertainment panel plus three additional trees from each of the core population sites in Limpopo, KwaZulu‐Natal and Eastern Cape Provinces (Appendix A: Figure A5). Samples were processed in quadruplicate and a > 97% call rate was obtained. The median genotyping error rate between replicates for the *Greyia* 23 SNP Type assay was 10%.

The *Greyia* 23 SNP Type assay data was then analyzed on the 73 wild‐growing *Greyia* trees sampled across the population range. Overall, after dereplication (i.e., retaining one of the quadruplicates with the least missing data), the SNP Type assay panel recovered full SNP genotypes for the 73 wild‐growing trees with a genotyping call rate of 98%. However, it failed to recover complete genotypes from the closely related *Melianthus* spp. and *Bersama lucens* used as outgroups (genotyping rate < 0.4), indicating that the novel *Greyia* 23 SNP Type assay is likely genus‐specific to *Greyia*.

All 23 SNP Type assays were polymorphic over all the 73 wild‐growing *Greyia* trees (Table 2). *PIC* ranged from 0.167 to 0.375, and the *H*_O_ and *H*_S_ ranged from 0.151 to 0.863 and 0.186 to 0.494, respectively. The 23 SNP Type assay data were first analyzed with an allele sharing matrix (ASM) to assign each of the 73 wild‐growing *Greyia* trees to a species cluster. This was successful for 71 of the trees since most shared > 99% (and at least 80%) genotypic information with one of the ascertainment panel species clusters (Appendix A: Table A1).

**TABLE 2:** Genetic diversity summary statistics of SNP Type assays applied to wild‐growing *Greyia* trees.

SNP type assay	All wild‐growing *Greyia* trees					Cluster I (“Sutherlandii”)				Cluster II (“Radlkoferi”)				Cluster III (“Flanaganii”)			
	*N*	*MAF*	*H*_O_	*H*_S_	*PIC*	*N*	*H*_O_	*H*_S_	*PIC*	*N*	*H*_O_	*H*_S_	*PIC*	*N*	*H*_O_	*H*_S_	*PIC*
*Greyia*_3RAD_D1_16417.28	73	0.445	0.836	0.497	0.372	43	0.953	0.505	0.374	20	1.000	0.513	0.375	10	0.00	0.00	0.000
*Greyia*_3RAD_D1_17901.48	72	0.194	0.306	0.315	0.264	42	0.476	0.433	0.336	20	0.050	0.050	0.048	10	0.10	0.10	0.090
*Greyia*_3RAD_D1_20015.167	73	0.486	0.151	0.503	0.375	43	0.256	0.291	0.247	20	0.000	0.000	0.000	10	0.00	0.00	0.000
*Greyia*_3RAD_D1_16438.7	73	0.144	0.014	0.248	0.216	43	0.023	0.023	0.023	20	0.000	0.000	0.000	10	0.00	0.00	0.000
*Greyia*_3RAD_D1_18402.102	73	0.233	0.055	0.360	0.293	43	0.000	0.000	0.000	20	0.200	0.262	0.222	10	0.00	0.00	0.000
*Greyia*_3RAD_D1_20179.46	73	0.473	0.616	0.502	0.374	43	0.721	0.479	0.361	20	0.450	0.358	0.288	10	0.50	0.39	0.305
*Greyia*_3RAD_D1_16444.229	73	0.103	0.151	0.186	0.167	43	0.000	0.000	0.000	20	0.550	0.481	0.359	10	0.00	0.00	0.000
*Greyia*_3RAD_D1_19009.73	73	0.226	0.178	0.352	0.289	43	0.302	0.479	0.361	20	0.000	0.000	0.000	10	0.00	0.00	0.000
*Greyia*_B1rSNP_its2_01	73	0.240	0.342	0.367	0.298	43	0.279	0.243	0.211	20	0.450	0.358	0.288	10	0.40	0.44	0.332
*Greyia*_3RAD_D1_16938.173	73	0.425	0.822	0.492	0.369	43	0.814	0.488	0.366	20	0.950	0.512	0.374	10	0.60	0.44	0.332
*Greyia*_3RAD_D1_19082.71	73	0.493	0.521	0.503	0.375	43	0.372	0.492	0.368	20	0.650	0.450	0.342	10	0.90	0.52	0.372
*Greyia*_B1rSNP_its2_02	73	0.308	0.507	0.429	0.336	43	0.814	0.506	0.375	20	0.100	0.097	0.090	10	0.00	0.00	0.000
*Greyia*_3RAD_D1_16946.51	72	0.493	0.097	0.503	0.375	43	0.163	0.260	0.224	19	0.000	0.000	0.000	10	0.00	0.00	0.000
*Greyia*_3RAD_D1_19227.91	73	0.144	0.014	0.248	0.216	43	0.023	0.023	0.023	20	0.000	0.000	0.000	10	0.00	0.00	0.000
*Greyia*_B1rSNP_its2_03	73	0.432	0.863	0.494	0.370	43	1.000	0.506	0.375	20	1.000	0.513	0.375	10	0.00	0.00	0.000
*Greyia*_3RAD_D1_17188.43	72	0.243	0.042	0.371	0.300	42	0.000	0.000	0.000	20	0.150	0.224	0.195	10	0.00	0.00	0.000
*Greyia*_3RAD_D1_19287.52	68	0.404	0.426	0.485	0.366	38	0.658	0.464	0.353	20	0.200	0.262	0.222	10	0.00	0.00	0.000
*Greyia*_B1rSNP_m1tK_01	73	0.144	0.123	0.248	0.216	43	0.093	0.131	0.121	20	0.000	0.000	0.000	10	0.50	0.39	0.305
*Greyia*_3RAD_D1_17357.154	70	0.407	0.414	0.486	0.366	42	0.262	0.503	0.374	19	0.947	0.512	0.374	9	0.00	0.00	0.000
*Greyia*_3RAD_D1_19922.176	72	0.292	0.111	0.416	0.328	43	0.186	0.506	0.375	19	0.000	0.000	0.000	10	0.00	0.00	0.000
*Greyia*_B1rSNP_trnLH_01	72	0.236	0.028	0.363	0.296	42	0.048	0.488	0.366	20	0.000	0.000	0.000	10	0.00	0.00	0.000
*Greyia*_3RAD_D1_17371.56	73	0.199	0.397	0.321	0.268	43	0.047	0.046	0.044	20	0.850	0.501	0.369	10	1.00	0.53	0.375
*Greyia*_3RAD_D1_19983.195	67	0.224	0.239	0.350	0.287	40	0.350	0.292	0.247	19	0.053	0.053	0.050	8	0.125	0.125	0.110
Mean	72	0.304	0.315	0.393	0.309	42	0.341	0.311	0.240	20	0.330	0.224	0.173	10	0.180	0.130	0.097

*Note:* Column headings refer to number of samples (*N*), minor allele frequency (*MAF*), observed heterozygosity (*H*_O_), gene diversity (*H*_S_), and polymorphic information content (*PIC*). Clusters I‐III refer to the species assignments of the wild‐growing *Greyia* trees based on the genetic clusters determined from the Allele Sharing Matrix calculated in Microsatellite Toolkit of GenAlEx v. 6.503.

**FIGURE 2:**
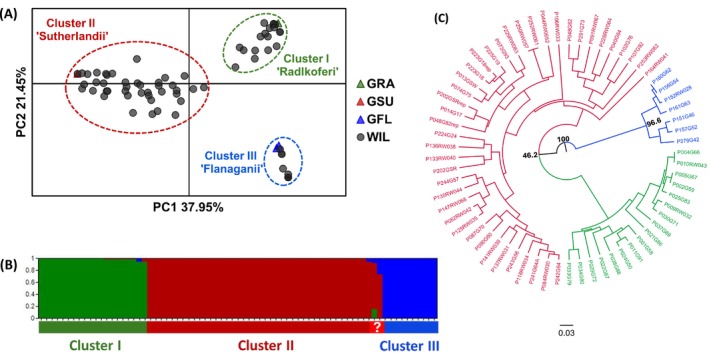
Wild‐growing *Greyia* trees form three genetic clusters with 23 SNP Type assay. (A) Principle Components Analysis with the 23 SNP data show that the 73 wild‐growing trees form three *Greyia* species clusters: Cluster I—“Radlkoferi,” Cluster II—“Sutherlandii,” Cluster III—“Flanaganii.” The *G. radlkoferi* , *G. sutherlandii*, *G. flanaganii* species‐representative ascertainment panel members are indicated by triangles: GRA (green); GSU (red); GFL (blue). The other wild‐growing *Greyia* trees are indicated by gray circles. PC1 and PC2 describe 37.95% and 21.45% of the variation, respectively. (B) Admixture analysis of the same SNP dataset as (A) shows that the 73 wild‐growing *Greyia* trees form the same three clusters of inferred ancestory (*K* = 3) visualized with an ADMIXTURE bar plot. (C) UPGMA dendrogram calculated from the matrix of shared alleles of the same SNP dataset as (A) groups the 73 wild‐growing trees into three *Greyia* species clades indicated by the color coding: GRA (green); GSU (red); GFL (blue). *Greyia* tree IDs correspond to the trees listed in the Appendix A: Table A1. The scale bar illustrates the amount of genetic distance expressed as the proportion of differing alleles.

**FIGURE A6:**
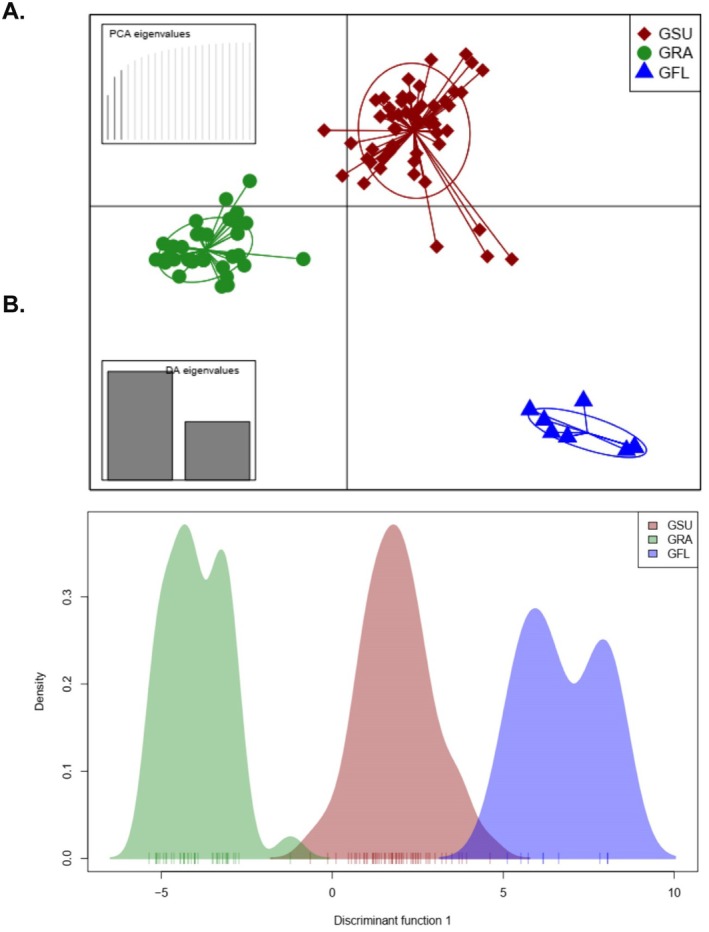
Discriminant Analysis of Principle Components (DAPC) shows that wild‐growing Greyia trees form three genetic clusters. (A) DAPC plot generated with the 23 SNP Type^TM^ assay data of the 73 wild‐growing trees illustrates the three *Greyia* species clusters: Cluster I—“Radlkoferi” (GRA, green circles), Cluster II—“Sutherlandii” (GSU, red diamonds), Cluster III—“Flanaganii” (GFL, blue triangles). (B) Density plot of the DAPC illustrating the three species clusters in the same color coding as A.

Additional genetic analyses of the 23 SNP Type assay data corroborated the assignment of each tree to one of the three species clusters. PCoA analysis confirmed that the 73 trees formed three major genetic clusters that corresponded with the ascertainment panel species groups (Cluster I “Sutherlandii”, Cluster II “Radlkoferi” and Cluster III “Flanaganii”; Figure 2A). The postprocessing of the ADMIXTURE results based on lowest average CV score across replicates identified *K* = 3 as the most likely number of genetic clusters, corresponding to the PCoA results (Figure 2B). An allele sharing distance‐based UPGMA dendrogram clearly assigned the 73 *Greyia* trees to the three species genetic clusters (Figure 2C). The DAPC analysis also differentiated the wild‐growing Greyia trees into three groups (Appendix A: Figure A6). However, finer‐scale population structure patterns within Cluster I “Sutherlandii” were detected; therefore, individual assignment based on genetic clusters (without prior group info) required *k* = 3 to be enforced (Appendix A: Figure A6). There were 24 *Greyia* trees that could not be identified in the field based on leaf and flower morphology, which were now assigned to either the “Radlkoferi” (7 trees) or “Sutherlandii” cluster (17 trees) (Appendix A: Table A1). Furthermore, morphological and 23‐SNP DNA‐based identifications were matched for 26 *G. sutherlandii*, 13 *G. radlkoferi* , and for all 10 *G. flanaganii* wild‐growing trees (Appendix A: Table A1).

**FIGURE 3:**
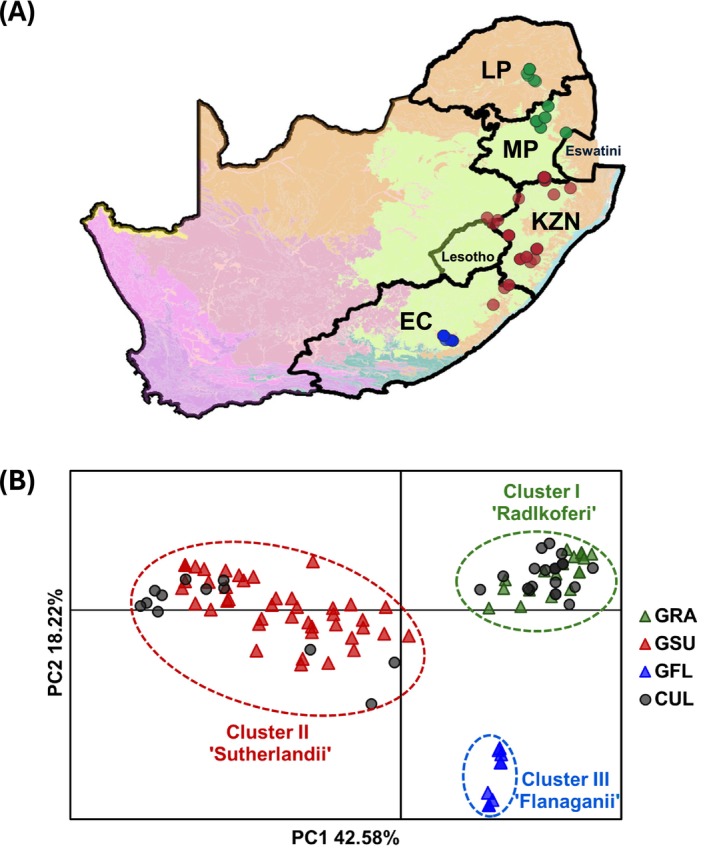
Biogeographical distribution of wild‐growing *Greyia* plants and identification of cultivated *Greyia* plants with 23 SNP Type assay. (A) Map of South Africa with locations of 73 wild‐growing *Greyia* trees, color coded by the species assignment from the 23 SNP Type assay. Trees identified as *G. radlkoferi* (green) are located in Limpopo and Mpumalanga provinces; *G. sutherlandii* (red) trees in southern Mpumalanga, KwaZulu‐Natal, and north‐east Eastern Cape provinces, and *G. flanaganii* (blue) in the Eastern Cape province. (B) Principle Components Analysis with the 23 SNP data show that the 33 cultivated trees cluster in one of the three *Greyia* species clusters: Cluster I—“Radlkoferi,” Cluster II—“Sutherlandii,” Cluster III—“Flanaganii.” The *G. radlkoferi* , *G. sutherlandii*, *G. flanaganii* wild‐growing trees are indicated by triangles: GRA (green); GSU (red); GFL (blue). The cultivated *Greyia* trees are indicated by gray circles. PC1 and PC2 describe 42.58% and 18.22% of the variation, respectively. The species assignments, identities and location of the cultivated trees are given in Appendix A: Table A1.

Overall, the species composition of the 73 wild‐growing *Greyia* trees was determined to be 43 *G. sutherlandii* (Cluster I “Sutherlandii”), 20 *G. radlkoferi* (Cluster II “Radlkoferi”), and 10 *G. flanaganii* (Cluster III “Flanaganii”). Furthermore, when the distribution of the *Greyia* trees was plotted on a map of South Africa, a clear phylogeographic structure was observed. As shown in Figure 3A, the Cluster 2 (“Radlkoferi”) trees were located in Limpopo and the northern part of Mpumalanga, the Cluster 1 (“Sutherlandii”) trees were in the southern part of Mpumalanga, KwaZulu‐Natal, and the eastern part of Eastern Cape. Cluster 3 (“Flanaganii”) trees were west of the Kei river in Eastern Cape, as expected (Figure 3A). Interestingly, the two trees that had < 80% shared genotype data with an ascertainment panel species had intermediate geographical locations, indicating incomplete divergence. These two exceptions were P164_RW041 from the Eastern Cape which shared 73% and 27% genotypic information with *G. sutherlandii* and *G. flanaganii*, respectively, and P102_G76 from the KwaZulu‐Natal/Mpumalanga border with 68% and 23% genotypic sharing with *G. radlkoferi*, respectively (Appendix A: Table A1).

### 3.4 Case Study—Identification of *Greyia* Orchard Trees

**FIGURE A7:**
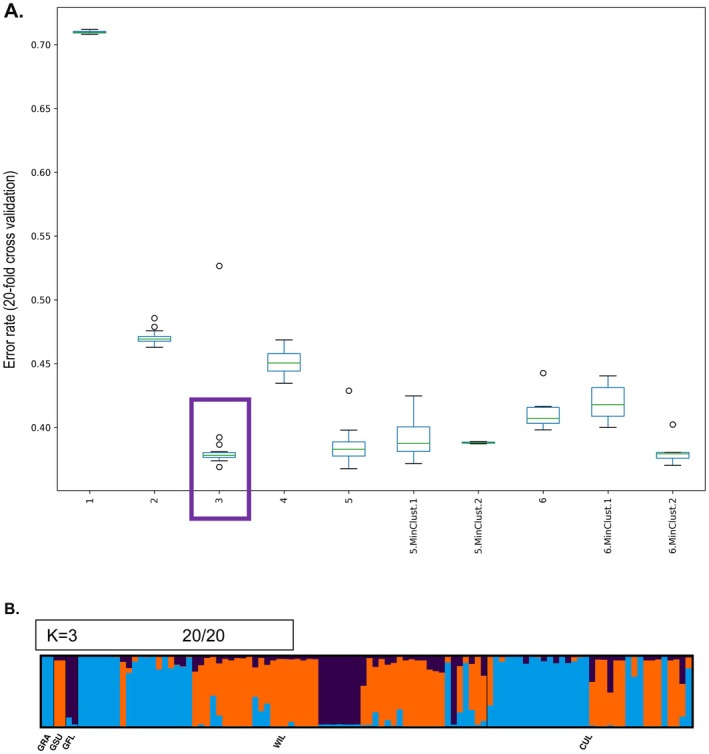
Admixture analysis indicates that cultivated and wild‐growing *Greyia* trees form three genetic clusters with the 23 SNP Type^TM^ assay. (A) ADMIXTURE error rates from 20‐fold cross‐validation tests to determine the optimal number of clusters (ancestral populations (K)) based on the 23 SNP data of 33 cultivated and 73 wild‐growing *Greyia* trees. The purple box illustrates that the lowest error rates were obtained for *K* = 3. Each box indicates the interquartile range, while the horizontal line within the box shows the median. Whisker bars show the maximum and minimum values, with outliers shown with open circles. (B) STRUCTURE plot illustrating that *K* = 3 was the optimal number of inferred ancestral populations for the SNP dataset of cultivated and wild‐growing *Greyia* trees. The ascertainment panel trees are labelled GRA, GSU, and GFL. The wild‐growing and cultivated trees are labelled as WIL and CUL, respectively.

*Greyia* trees are being cultivated in orchards in Gauteng province of South Africa to provide a source of leaf material for herbal remedies for hyper‐pigmentation. However, these trees were sourced from local nurseries and therefore the natural origin and species identities are not known. The *Greyia* 23 SNP Type assay was applied to 33 trees from two production orchards. All of these trees could be assigned to either Cluster I “Sutherlandii” or Cluster II “Radlkoferi,” as illustrated by PCoA analysis (Figure 3B, Appendix A: Table A1). Furthermore, Admixture analysis of the 23 SNP dataset for the 73 wild‐growing trees (including the ascertainment panel trees) and the 33 cultivated trees showed that *K* = 3 resulted in the lowest 20‐fold cross validation error rates (Appendix A: Figure A7A). The ADMIXTURE plot illustrated that the cultivated trees fell into either the *G. sutherlandii* or *G. radlkoferi* genotype groups (Appendix A: Figure A7B). The Allele Sharing Matrix (ASM) analysis assigned all 10 trees at Mothong Cultural Heritage orchard, Mamelodi to “Radlkoferi” with > 99% sharing of the genotypic information with the *G. radlkoferi* ascertainment panel members (Appendix A: Table A1). Interestingly, all of these plants were difficult to identify based on field morphology as they had a mix of wooly, hairy and glabrous leaves (Appendix A: Table A1). Ing et al. (2020) also reported morphological variation for trees in this orchard. The trees at the Future Africa orchard and Cycad and Indigenous Plant Nursery at Manie van der Schijff Botanical Gardens of the University of Pretoria were made up of 11 “Radlkoferi” and 12 “Sutherlandii” types. Interestingly, 10 of the “Radlkoferi” plants had > 99% genotypic information shared with *G. radlkoferi* ascertainment panel members, whereas one plant had only 85% with *G. radlkoferi* and 15% with the *G. flanaganii* panels. Nine of the Future Africa orchard plants had > 99% genotypic sharing with *G. sutherlandii* ascertainment panel members, whereas three plants showed significant proportions of *G. flanaganii* genotype (namely P264_RW024–78% GSU/22% GFL, P290_G35–70% GSU/30% GFL and P267_G09–62% GSU/38% GFL) (Appendix A: Table A1). The provenance and breeding history of these plants is unknown. These results could be explained by the possibility that they were derived from seed of crosses in the source nursery(s). This would point to interspecific crosses being possible between *Greyia* species. Alternatively, they were derived from cuttings of wild plants from populations not sampled in this study.

### 3.5 Comparison to Other Studies of RADseq Based SNP Discovery in Plants

The choice of restriction enzymes for RADseq library construction is an important consideration to ensure the capture of polymorphic genomic regions from the target plant taxa (Bayona‐Vasquez et al. 2019). Generally, this must be determined empirically as it may depend on genome size, coding gene distribution, GC content, and other factors. The most commonly used restriction enzymes for plant RADseq and other reduced representation methods are *Eco*RI and *Msp*I, as demonstrated in potato and Arabidopsis (Jiang et al. 2016). In our study on the tree genus *Greyia* in the order *Geraniales*, *Eco*RI‐*Xba*I RAD fragments were targeted in the 3RAD approach that included *Nhe*I to prevent adapter dimers (Bayona‐Vasquez et al. 2019). This enzyme combination was used in the 3RAD proof of concept with the plant genus *Wisteria* (Bayona‐Vasquez et al. 2019), where libraries from 24 plants resulted in 30,029 RAD loci, which were filtered to 5820 polymorphic SNPs.

A study in lettuce combined SNP discovery using reduced genome representation followed by design of a small SNP panel (Park et al. 2022), similar to our approach in Greyia (Appendix A: Figure A1). The aim was to differentiate 90 lettuce cultivars with SNP discovery based on using the *ApeK1* restriction enzyme to make libraries for Illumina sequencing (Park et al. 2022). After filtering, they obtained 17,877 SNPs which clustered the lettuce genotypes into three main groups using similar population genetics approaches as our study (Park et al. 2022). The SNPs were filtered using PIC values into subsets of 192 SNPs and eventually 24 SNPs, which recapitulated the three genetic groups seen with the larger SNP set. The final 24 SNP set was able to identify 88% of the lettuce cultivars (Park et al. 2022).

In the threatened Dragonhead plant, RADseq was used for discovery of 96 SNPs across the distribution range in Norway, which were subsequently used to screen additional populations for conservation planning (Kleven et al. 2019). Microfluidics or KASP‐based systems with small SNP panels have previously been used for plant identifications, for example *Brassica* (10 SNPs), *Lasthenia* (California goldfields) (44 SNPs), *Coffea* (55 SNPs), and *Dracocephalum ruyschiana* (96 SNPs) (Jo et al. 2022; Torres‐Martínez and Emery 2016; Zhou et al. 2016; Kleven et al. 2019).

Implementation of the final 23 SNP panel for *Greyia* species identification was carried out on a microfluidics system (Appendix A: Figure A1). This holds several advantages if the Biomark HD instrumentation is available, since very small quantities of gDNA are required per reaction. Multilocus genotyping with a small SNP panel on a microfluidics system has advantages over standard plastid barcodes since a larger number of alleles can be screened in a single assay (Park et al. 2022), in contrast to individual Sanger sequencing of each plastid barcode (Howard et al. 2020). Moreover, in our hands, the 23 SNP Type assay on the microfluidics system for 72 *Greyia* tree samples was calculated to require half the cost and half the time compared to standard sequence‐based barcoding for four gene regions, with the added limitation as reported in Botha et al. (2026) that the barcodes did not differentiate the *Greyia* species.

### 3.6 Implications for Conservation and Future Genetic Studies of *Greyia*

Overall, this work has advanced knowledge of the genetics of the tree genus *Greyia* from southern Africa. Prior DNA barcoding attempts of *Greyia* had yielded insufficient polymorphisms to differentiate the three *Greyia* species (Palazzesi et al. 2012; Botha et al. 2026). In this work, 73 wild‐growing *Greyia* trees across the population range from the Eastern Cape, KwaZulu‐Natal, Free State, Mpumalanga, and Limpopo Provinces were sampled. Genetic relationships between these trees were established using a set of 23 genome‐wide SNPs made up of 21 nuclear and two plastid SNPs which differentiate individuals into three groups corresponding to geographically separate representatives of the three species, *G. radlkoferi* , *G. sutherlandii*, and *G. flanaganii* (Figure 3A).

Prior to this study, herbarium records and field guides showed overlap of the distributions of *G. radlkoferi* and *G. sutherlandii* in Mpumalanga Province (De la Cruz 2016), however, our 23 SNP Type assay grouped northern Mpumalanga trees as *G. radlkoferi* and southern Mpumalanga trees as *G. sutherlandii* (Figure 3A). Both the RADseq data from eight representative individuals and the *Greyia* 23 SNP Type assay data for all 73 wild‐growing trees showed that *G. flanaganii* was the most genetically distinct of the three species. This is consistent with published ecological, morphological, and genetic data (Steyn et al. 1999; Botha et al. 2026).

We acknowledge some limitations the *Greyia* 23 SNP Type assay as a species identification tool and that it is not suitable for in‐depth intraspecific biogeographic studies in *Greyia*, such as determining population structure and demography. First, it is based on defining a “genetic type” for each of the three species based on the ascertainment panel of eight trees selected from locations close to that of the original type specimens used to describe each species. However, this may not reflect the true species types or capture the complete diversity. *Greyia* trees tend to occupy specific ecological niches in mountainous regions which may not be contiguous in space, leading to possible genetic isolation of some populations. Similar fragmented landscapes led to distinct populations of three endemic species of *Mammillaria* cacti in Mexico (Lázaro‐Castellanos et al. 2022). In future work, extending the population genetics approach by conducting genome‐wide SNP analysis from 3RAD data of all individuals from a deeper sampling pool (i.e., all 73 trees in this study plus individuals from additional sites) would shed light on the number of genetic groups. This would also facilitate demarcation of the current three species or conservation units (CUs) into sub‐populations or “evolutionarily sustaining conservation units” (ECSUs) that may need different conservation management approaches.

Ongoing research is testing whether the *Greyia* species can form fertile F1 hybrids, meaning that there may not be reproductive barriers for the *Greyia* genus. The current study will facilitate such a study as eight of the 21 nuclear SNPs had homozygous “species‐diagnostic” SNPs in all eight of the ascertainment panel trees (Supplementary Dryad dataset). These can be developed into PCR assays to screen F1 progeny. In addition, this work will facilitate identification of parental plants for crossbreeding by selecting cuttings of wild‐growing trees or other trees at the Manie van der Schijff Botanical Gardens, University of Pretoria that have > 99% genotypic identity with the ascertainment panel members. There are many precedents for hybridization within a plant genus such as between *Aloe* species (Cousins and Witkowski 2012), although some Aloes appear to partition pollinators in time or floral attractiveness to prevent hybridization in nature (Botes et al. 2008). The dataset of 47,356 genome‐wide SNPs from the 3RAD will also be a useful future tool for genetic mapping of traits in *Greyia* spp. if crossbreeding is possible between one or more of the three *Greyia* species. This approach, however, could be hampered by the long generation time of *Greyia* trees before reaching flowering maturity in each generation.

The 23 SNP panel has not only value for both in situ conservation but also *ex situ* conservation since in the case study it was shown that the panel could be applied to identify the correct *Greyia* species in orchards to be used for medicinal purposes. There are currently efforts by communities in Gauteng Province, such as the Mothong Cultural Heritage orchard sampled in this study and also the Eastern Cape Province to promote the growing of *Greyia* orchards for medicinal purposes. Furthermore, the striking red flowers of *Greyia* underpin their horticultural potential recognized as early as the late 1800's in Europe (Killick and Kimpton 1977). Verification of species genotypes with this DNA assay will facilitate the ex‐situ conservation of each species in botanical and private gardens.

### 3.7 Conclusions

Mis‐identification of morphologically similar source plants is an ongoing challenge in phytomedicine as well as quality control of herbal products (Street et al. 2008). In this work, this challenge was addressed by developing a DNA diagnostic (23 SNP panel) for differentiating species of the medicinally important tree genus *Greyia*. This will ensure correct assignment of *Greyia* species to the Red List of South African Plants administered by the South African National Biodiversity Institute. Currently *G. flanaganii* is of the most conservation concern of the three, as it is listed as Rare on account of its limited distribution (https://redlist.sanbi.org/search.php?sppsearch=greyia). The SNP assay will also be valuable for provenance certification of *Greyia*‐derived herbal remedies and ensure sustainable harvesting in line with the National Environmental Management Biodiversity Act of South Africa.

The SNP discovery and SNP assay methodology developed in this work (Appendix A: Figure A1) can be applied to other medicinal and nonmodel plants which lack genomic resources. It is especially useful for recently diverged taxa where standard DNA barcodes have insufficient polymorphisms. The 3RAD method was applied to a small number of diverse *Greyia* genotypes for SNP discovery, followed by population genetic approaches to filter down to a small panel of diagnostic SNPs for high‐throughput genotyping. As a proof of concept, the 23‐SNP DNA diagnostic was applied to cultivated *Greyia* trees of unknown identity in production orchards. The 23‐SNP assay was also used to survey the distribution of wild‐growing *Greyia* trees across a 1000 km transect in South Africa, and resulted in three species‐specific distribution ranges of *G. radlkoferi* , *G. sutherlandii*, and *G. flanaganii* from north to south, respectively.

## Author Contributions

**Iné Botha:** data curation (equal), formal analysis (lead), investigation (lead), methodology (supporting), resources (supporting), visualization (equal), writing – original draft (equal). **Simo N. Maduna:** conceptualization (supporting), data curation (equal), formal analysis (lead), investigation (equal), methodology (lead), software (lead), supervision (supporting), validation (equal), visualization (equal), writing – review and editing (equal). **Namrita Lall:** funding acquisition (supporting), resources (supporting), supervision (supporting), writing – review and editing (supporting). **Snorre B. Hagen:** funding acquisition (supporting), project administration (supporting), resources (supporting), writing – review and editing (supporting). **Dave K. Berger:** conceptualization (lead), data curation (equal), funding acquisition (lead), investigation (equal), project administration (lead), resources (equal), supervision (lead), visualization (supporting), writing – review and editing (equal).

## Funding

Funding for this research was provided by the Norwegian Institute of Bioeconomy Research (NIBIO) to SM, and by the National Research Foundation South Africa (NRF) Foundational Biodiversity Information Programme grant (FBIS2204041924) to D.K.B., an Oppenheimer Memorial Trust (OMT) Sabbatical Award to D.K.B. (OMT Ref. 2023‐1431), the NRF SARChI grant (SARCI150227114490) and DSI Funding (DST/CON 0024‐2015) to N.L., and a NRF SARChI grantholder‐linked scholarship to I.B. (98334). The sequencing service was provided by the Norwegian Sequencing Centre (www.sequencing.uio.no), a national technology platform hosted by the University of Oslo and supported by the “Functional Genomics” and “Infrastructure” programs of the Research Council of Norway and the Southeastern Regional Health Authorities. DNA extraction of silica dried *Greyia* leaf samples was done using the oktopure automated DNA extraction system at the Precision Tree Breeding Platform of the Forest Molecular Genetics Programme, University of Pretoria.

## Conflicts of Interest

The authors declare no conflicts of interest.

## Supporting information


**Data S1:** ece373412‐sup‐0001‐DataS1.pdf.

## Data Availability

Raw, demultiplexed sequencing data is available from the NCBI Sequence Read Archive (SRA) accession number PRJNA1434046 (SRR37525841–SRR37525856). The SNP genotypic dataset and bioinformatics workflow used in the current study is available on Dryad Repository (https://doi.org/10.5061/dryad.1ns1rn97b).

## Appendix A

**TABLE A1:** *Greyia* species assignments for wild‐growing and cultivated trees based on Greyia 23 SNP Type^TM^ assay.

Tree ID^a^	23 SNP genetic clusters^b^				Field Morphology^d^			Provenance	Province	QDS	Altitude (m)
	GRA	GSU	GFL	Species assignment^c^	Leaf	Flower	Species assignment^e^				
P001_G58	0.995	0.002	0.003	GRA	Woolly	Compact	GRA	wild‐growing	Limpopo	2330CC	1079
P002_G59	0.996	0.002	0.002	GRA	Woolly	Compact	GRA	wild‐growing	Limpopo	2330CC	1068
P003_G65A	0.995	0.002	0.003	GRA	Woolly	Compact	GRA	wild‐growing	Limpopo	2330CC	1090
**P004_G66**	0.997	0.001	0.002	GRA	Woolly	Compact	GRA	wild‐growing	Limpopo	2330CC	1055
**P005_G67**	0.996	0.002	0.002	GRA	Woolly	Compact	GRA	wild‐growing	Limpopo	2330CC	1038
P011_G91	0.998	0.001	0.001	GRA	Woolly	Compact	GRA	wild‐growing	Limpopo	2329DD	1236
P007_G90	0.996	0.002	0.002	GRA	Hairy	Compact	*Greyia* sp.	wild‐growing	Limpopo	2329DD	1516
P009_RW032	0.996	0.002	0.002	GRA	Hairy	Compact	*Greyia* sp.	wild‐growing	Limpopo	2430AA	1148
P010_RW043	0.996	0.002	0.002	GRA	Woolly	Compact	GRA	wild‐growing	Limpopo	2430AA	1275
P030_G71	0.996	0.002	0.002	GRA	Glabrous	Compact	*Greyia* sp.	wild‐growing	Mpumalanga	2430DC	1787
P029_G72	0.906	0.094	0	GRA	Glabrous	Compact	*Greyia* sp.	wild‐growing	Mpumalanga	2430DC	1787
P024_G50	0.998	0.001	0.001	GRA	Woolly	Compact	GRA	wild‐growing	Mpumalanga	2530AC	1847
P037_G89	0.999	0	0.001	GRA	Woolly	Compact	GRA	wild‐growing	Mpumalanga	2530AD	1394
P025_G83	0.998	0.001	0.001	GRA	Woolly	Compact	GRA	wild‐growing	Mpumalanga	2530AD	1342
**P023_G61**	0.996	0.002	0.002	GRA	Woolly	Compact	GRA	wild‐growing	Mpumalanga	2530AD	1503
P038_G88	0.998	0.001	0.001	GRA	Glabrous	unknown	*Greyia* sp.	wild‐growing	Mpumalanga	2530BC	1668
P034_G80	0.999	0	0.001	GRA	Woolly	Compact	GRA	wild‐growing	Mpumalanga	2530CB	1565
P033_G79	0.941	0.059	0	GRA	Woolly	Compact	GRA	wild‐growing	Mpumalanga	2530CB	1425
P021_G86	0.999	0.001	0	GRA	unknown	Compact	*Greyia* sp.	wild‐growing	Mpumalanga	2531CC	1453
P022_G87	0.997	0.002	0.001	GRA	unknown	Compact	*Greyia* sp.	wild‐growing	Mpumalanga	2531CC	1466
P291_G73	0	0.974	0.026	GSU	Glabrous	unknown	*Greyia* sp.	wild‐growing	Mpumalanga	2528CD	1560
P040_G84	0	0.999	0.001	GSU	Woolly	unknown	*Greyia* sp.	wild‐growing	Mpumalanga	2530BC	1419
P013_GSW	0.001	0.999	0	GSU	Glabrous	Oblong	GSU	wild‐growing	Mpumalanga	2730AB	1832
P044_RW065.2	0	0.999	0.001	GSU	Glabrous	Oblong	GSU	wild‐growing	Mpumalanga	2730AB	1539
P229_RW063	0.001	0.999	0	GSU	Glabrous	Oblong	GSU	wild‐growing	Mpumalanga	2730AB	1927
P223_G18	0.001	0.998	0.001	GSU	Glabrous	Oblong	GSU	wild‐growing	Mpumalanga	2730AB	1832
P223_G18	0.001	0.998	0.001	GSU	Glabrous	Oblong	GSU	wild‐growing	Mpumalanga	2730AB	1832
P225_G19	0.001	0.998	0.001	GSU	Glabrous	Oblong	GSU	wild‐growing	Mpumalanga	2730AB	1814
P014_G17	0.001	0.997	0.002	GSU	Glabrous	Oblong	GSU	wild‐growing	Mpumalanga	2730AB	1950
P228_RW064	0.002	0.995	0.003	GSU	Glabrous	Oblong	GSU	wild‐growing	Mpumalanga	2730AB	1899
P048_G82	0.003	0.994	0.003	GSU	Glabrous	Oblong	GSU	wild‐growing	Mpumalanga	2730AB	1993
P048_G82	0.003	0.994	0.003	GSU	Glabrous	Oblong	GSU	wild‐growing	Mpumalanga	2730AB	1993
P224_G24	0	0.999	0.001	GSU	Glabrous	Oblong	GSU	wild‐growing	Mpumalanga	2730BA	1840
P202_GSR	0.001	0.998	0.001	GSU	Glabrous	Oblong	GSU	wild‐growing	Mpumalanga	2730BA	1830
P202_GSR	0.001	0.998	0.001	GSU	Glabrous	Oblong	GSU	wild‐growing	Mpumalanga	2730BA	1830
P074_G75	0.001	0.998	0.001	GSU	Woolly	Oblong	*Greyia* sp.	wild‐growing	Mpumalanga	2730BC	1700
P073_G93	0.127	0.873	0	GSU	Hairy	Oblong	*Greyia* sp.	wild‐growing	Mpumalanga	2730BC	1673
P107_G92	0.133	0.867	0	GSU	Hairy	unknown	*Greyia* sp.	wild‐growing	KwaZulu‐Natal	2730BC	1660
P106_RW033	0.002	0.997	0.001	GSU	Woolly	unknown	*Greyia* sp.	wild‐growing	KwaZulu‐Natal	2730DD	1335
P102_G76	0.23	0.683	0.087	GSU	unknown	unknown	*Greyia* sp.	wild‐growing	KwaZulu‐Natal	2731CB	1450
P118_RW034	0.002	0.996	0.002	GSU	Glabrous	unknown	*Greyia* sp.	wild‐growing	KwaZulu‐Natal	2729DC	1799
P135_RW044	0.003	0.995	0.002	GSU	Glabrous	Oblong	GSU	wild‐growing	KwaZulu‐Natal	2828DB	1487
P125_RW035	0.002	0.995	0.003	GSU	Glabrous	unknown	*Greyia* sp.	wild‐growing	KwaZulu‐Natal	2829CA	1682
**P242_G64**	0.001	0.998	0.001	GSU	Glabrous	Oblong	GSU	wild‐growing	KwaZulu‐Natal	2929AB	1585
**P245_G65**	0.001	0.998	0.001	GSU	Glabrous	Oblong	GSU	wild‐growing	KwaZulu‐Natal	2929AB	1702
P241_G64A	0.002	0.997	0.001	GSU	Glabrous	Oblong	GSU	wild‐growing	KwaZulu‐Natal	2929AB	1508
P244_G57	0.002	0.997	0.001	GSU	Glabrous	Oblong	GSU	wild‐growing	KwaZulu‐Natal	2929AB	1684
P243_G56	0.002	0.996	0.002	GSU	Glabrous	Oblong	GSU	wild‐growing	KwaZulu‐Natal	2929AB	1680
P249_RW058	0.001	0.998	0.001	GSU	Glabrous	Oblong	GSU	wild‐growing	KwaZulu‐Natal	2929DB	1337
P136_RW038	0.002	0.996	0.002	GSU	Glabrous	unknown	*Greyia* sp.	wild‐growing	KwaZulu‐Natal	2929DB	1342
P137_RW031	0.003	0.995	0.002	GSU	Glabrous	Oblong	GSU	wild‐growing	KwaZulu‐Natal	2929DD	1792
P141_RW039	0.003	0.995	0.002	GSU	Glabrous	Oblong	GSU	wild‐growing	KwaZulu‐Natal	2929DD	1751
**P090_G60**	0.003	0.995	0.002	GSU	Glabrous	Oblong	GSU	wild‐growing	KwaZulu‐Natal	2930AD	1032
P087_G70	0.001	0.998	0.001	GSU	Glabrous	Oblong	GSU	wild‐growing	KwaZulu‐Natal	2930AD	1021
P091_RW067	0.003	0.994	0.003	GSU	Woolly	Oblong	*Greyia* sp.	wild‐growing	KwaZulu‐Natal	2930AD	1030
P133_RW040	0.002	0.996	0.002	GSU	Glabrous	unknown	*Greyia* sp.	wild‐growing	KwaZulu‐Natal	2930CC	992
P147_RW068	0.002	0.995	0.003	GSU	Glabrous	unknown	*Greyia* sp.	wild‐growing	KwaZulu‐Natal	2930CC	1448
P250_RW057	0.001	0.997	0.002	GSU	Glabrous	unknown	*Greyia* sp.	wild‐growing	KwaZulu‐Natal	3029CB	1439
P252_RW061	0.001	0.997	0.002	GSU	Glabrous	unknown	*Greyia* sp.	wild‐growing	KwaZulu‐Natal	3029CB	1439
P084_RW030	0.002	0.997	0.001	GSU	Glabrous	Oblong	GSU	wild‐growing	Free State	2929DD	1799
P082_RW042	0.002	0.996	0.002	GSU	Glabrous	Oblong	GSU	wild‐growing	Free State	2828BC	1863
P253_RW062	0	0.954	0.046	GSU	Glabrous	unknown	*Greyia* sp.	wild‐growing	Eastern Cape	3029CB	1367
P164_RW041	0	0.73	0.27	GSU	Glabrous	Compact	*Greyia* sp.	wild‐growing	Eastern Cape	3128BD	890
P151_G46	0.001	0.001	0.998	GFL	Glabrous	urn‐shaped, petals not fused, hanging	GFL	wild‐growing	Eastern Cape	3227AB	732
P152_RW028	0.002	0.002	0.996	GFL	Glabrous	urn‐shaped, petals not fused, hanging	GFL	wild‐growing	Eastern Cape	3227AB	728
P153_RW029	0.001	0.001	0.998	GFL	Glabrous	urn‐shaped, petals not fused, hanging	GFL	wild‐growing	Eastern Cape	3227AB	753
P156_G54	0.001	0	0.999	GFL	Glabrous	urn‐shaped, petals not fused, hanging	GFL	wild‐growing	Eastern Cape	3227AB	697
P157_G52	0.002	0.001	0.997	GFL	Glabrous	urn‐shaped, petals not fused, hanging	GFL	wild‐growing	Eastern Cape	3227AD	1039
P158_G55	0.001	0.001	0.998	GFL	Glabrous	urn‐shaped, petals not fused, hanging	GFL	wild‐growing	Eastern Cape	3227BC	596
P159_G62A	0.002	0.002	0.996	GFL	Glabrous	urn‐shaped, petals not fused, hanging	GFL	wild‐growing	Eastern Cape	3227BC	608
**P160_G62**	0.002	0.001	0.997	GFL	Glabrous	urn‐shaped, petals not fused, hanging	GFL	wild‐growing	Eastern Cape	3227BC	605
**P161_G63**	0.001	0.001	0.998	GFL	Glabrous	urn‐shaped, petals not fused, hanging	GFL	wild‐growing	Eastern Cape	3227BC	594
P279_G42	0.003	0.003	0.994	GFL	Glabrous	urn‐shaped, petals not fused, hanging	GFL	wild‐growing	Eastern Cape	3227BC	950
P176_RW047	0.999	0	0.001	GRA	Glabrous	unknown	*Greyia* sp.	cultivated	Gauteng	2528CB	1416
P177_RW048	0.999	0	0.001	GRA	Woolly	unknown	*Greyia* sp.	cultivated	Gauteng	2528CB	1393
P178_RW049	0.999	0	0.001	GRA	Glabrous	unknown	*Greyia* sp.	cultivated	Gauteng	2528CB	1413
P179_RW050	0.999	0	0.001	GRA	Glabrous	unknown	*Greyia* sp.	cultivated	Gauteng	2528CB	1409
P174_RW045	0.998	0.001	0.001	GRA	Glabrous	unknown	*Greyia* sp.	cultivated	Gauteng	2528CB	1439
P180_RW052	0.998	0.001	0.001	GRA	Glabrous	unknown	*Greyia* sp.	cultivated	Gauteng	2528CB	1413
P182_RW054	0.998	0.001	0.001	GRA	Woolly	unknown	*Greyia* sp.	cultivated	Gauteng	2528CB	1413
P175_RW046	0.997	0.002	0.001	GRA	Woolly	unknown	*Greyia* sp.	cultivated	Gauteng	2528CB	1435
P183_RW053	0.997	0.001	0.002	GRA	Glabrous	unknown	*Greyia* sp.	cultivated	Gauteng	2528CB	1420
P184_RW055	0.995	0.002	0.003	GRA	Glabrous	unknown	*Greyia* sp.	cultivated	Gauteng	2528CB	1437
P195_RW069	0.999	0	0.001	GRA	Hairy	unknown	*Greyia* sp.	cultivated	Gauteng	2528CD	1412
P190_GRW	0.998	0.001	0.001	GRA	Hairy	unknown	*Greyia* sp.	cultivated	Gauteng	2528CD	1414
P197_RW059	0.998	0.001	0.001	GRA	Woolly	Compact	GRA	cultivated	Gauteng	2528CD	1383
P196_G36	0.997	0.002	0.001	GRA	Woolly	unknown	*Greyia* sp.	cultivated	Gauteng	2528CD	1408
P188_G4	0.996	0.001	0.003	GRA	Glabrous	Compact	GRA	cultivated	Gauteng	2528CD	1404
P198_RW060	0.995	0.002	0.003	GRA	Woolly	Compact	GRA	cultivated	Gauteng	2528CD	1382
P262_GR4362	0.999	0.001	0	GRA	Woolly	Compact	GRA	cultivated	Gauteng	2528CD	1399
P270_RW025	0.996	0.002	0.002	GRA	Woolly	unknown	*Greyia* sp.	cultivated	Gauteng	2528CD	1395
P272_GO2	0.995	0.002	0.003	GRA	Woolly	Compact	GRA	cultivated	Gauteng	2528CD	1396
P276_GO6	0.995	0.002	0.003	GRA	Woolly	Compact	GRA	cultivated	Gauteng	2528CD	1395
P271_GO1	0.85	0.15	0	GRA	Woolly	Compact	GRA	cultivated	Gauteng	2528CD	1401
P266_GO8	0.001	0.998	0.001	GSU	Glabrous	unknown	*Greyia* sp.	cultivated	Gauteng	2528CD	1396
P268_GO10	0.001	0.998	0.001	GSU	Glabrous	unknown	*Greyia* sp.	cultivated	Gauteng	2528CD	1394
P269_RW023	0.001	0.998	0.001	GSU	Glabrous	unknown	*Greyia* sp.	cultivated	Gauteng	2528CD	1399
P273_GO3	0.001	0.998	0.001	GSU	Hairy	Compact	*Greyia* sp.	cultivated	Gauteng	2528CD	1395
P274_GO4	0.001	0.998	0.001	GSU	Glabrous	Oblong	GSU	cultivated	Gauteng	2528CD	1397
P278_RW027	0.001	0.998	0.001	GSU	Glabrous	Oblong	GSU	cultivated	Gauteng	2528CD	1392
P277_RW026	0.002	0.997	0.001	GSU	Glabrous	Oblong	GSU	cultivated	Gauteng	2528CD	1396
P265_GO7	0.002	0.996	0.002	GSU	Glabrous	Oblong	GSU	cultivated	Gauteng	2528CD	1394
P275_GO5	0.002	0.996	0.002	GSU	Glabrous	Oblong	GSU	cultivated	Gauteng	2528CD	1396
P264_RW024	0	0.783	0.217	GSU	Glabrous	Oblong	GSU	cultivated	Gauteng	2528CD	1389
P267_GO9	0	0.624	0.376	GSU	Glabrous	unknown	*Greyia* sp.	cultivated	Gauteng	2528CD	1396
P290_G35	0	0.704	0.296	GSU	Glabrous	unknown	*Greyia* sp.	cultivated	Gauteng	2528CD	1407

*Note:* The location of each *Greyia* tree is given per Province in South Africa, and the Quarter Degree Square (QDS) also known as Quarter Degree Grid Cells commonly used in biodiversity studies in southern Africa (see https://thebdi.org/2019/06/27/quarter‐degree‐grid‐cells‐made‐simple/) Altitude is reflected as meters above sea level.

^a^ TreeID—the P number refers to a specific tree, and the second code refers to a lab code (DNA extraction). *Greyia* species ascertainment panel trees are marked in bold.

^b^ Fraction of alleles shared with each of the three *Greyia* species clusters (GRA = “Radlkoferi,” GSU = “Sutherlandii,” GFL = “Flanaganii”) determined from the Allele Sharing Matrix calculated in MICROSATELLITE TOOLKIT of genalex v. 6.503.

^c^ *Greyia* species assignment based on Allele Sharing Matrix.

^d^ Field morphology based on leaf type and flower shape.

^e^ *Greyia* species assignment based on field morphology traits.
