# Correction to “3RAD‐Guided SNP Discovery for Species Identification and Conservation of the Medicinal Southern African Tree Genus *Greyia* Hook. & Harv.”

**DOI:** 10.1002/ece3.73827

**Published:** 2026-06-08

Botha, I., S. N. Maduna, S. B. Hagen, N. Lall, and D. K. Berger. 2026. “3RAD‐Guided SNP Discovery for Species Identification and Conservation of the Medicinal Southern African Tree Genus Greyia Hook. & Harv.” *Ecology and Evolution* 16, no. 5: e73412. https://doi.org/10.1002/ece3.73412.

In the Acknowledgements section of the original article, several contributors were inadvertently omitted. The Acknowledgements should include the following text:

The authors acknowledge J Pretorius and the Buffelskloof Nature Reserve, the South African National Biodiversity Institute (SANBI)–Kirstenbosch National Botanical Garden, the Wildlife and Environment Society of South Africa (WESSA)–Umgeni Valley Nature Reserve, and Ohrigstad Dam Nature Reserve for providing sampling permits and field assistance.

We apologize for this error.

