# Supplementary material for: 3RAD‐Guided SNP Discovery for Species Identification and Conservation of the Medicinal Southern African Tree Genus Greyia Hook. & Harv
Source: Ecol Evol. 2026 May 5;16(5):e73412. doi: 10.1002/ece3.73412 (PMC13139959; doi:10.1002/ece3.73412)
Supplement: Supplementary file 1 — Data S1: ece373412‐sup‐0001‐DataS1.pdf. [file ECE3-16-e73412-s001.pdf]

3RAD-guided SNP discovery for species identification and conservation of the medicinal southern African tree genus *Greyia* Hook. & Harv.

Iné Botha[1,2], Simo N. Maduna[3,*], Snorre B. Hagen[3], Namrita Lall[1], Dave K. Berger[1,2*]

[1]Department of Plant and Soil Sciences, [2]Forestry and Agricultural Biotechnology Institute (FABI), University of Pretoria, Hatfield, 0002, South Africa

[3]Department of Ecosystems in the Barents Region, Svanhovd Research Station, Norwegian Institute of Bioeconomy Research (NIBIO), Svanvik, 9925, Norway

* *To whom correspondence should be addressed*:
S.N. Maduna (simo.maduna@nibio.no) and D.K. Berger (dave.berger@fabi.up.ac.za)

Table of contents:

**Extended Materials and Methods**	Page 1-3
**Figure S1**	Page 4-5
**Figure S2**	Page 6
**Supplementary References**	Page 6

Extended Materials and Methods

DNA Extraction protocol 1 (for 3RAD library construction)

Manual CTAB method to extract genomic DNA (gDNA) from *Greyia* leaf material (modified from (Botha et al. 2026).

1. Prepare CTAB buffer
   - -2% w/v CTAB
   - -100 mM Tris-HCl (pH 8)
   - -20 mM EDTA (pH 8)
   - -1.4 M NaCl
   - -80 mM Na_2SO_3
2. Activate the CTAB buffer by adding 5% polyvinylpyrrolidone (PVP-10) and 2% v/v β-mercaptoethanol
3. Incubate the activated CTAB buffer at 65 °C.
4. Weigh out 2g of leaf material. Note: DNA quality and yield is highest if young leaf material (newly emerged flush) is used. Leaf material collected in the field should be immediately placed into ziplock bags with silica gel beads (Merck, catalogue # 1.07735). Alternatively, DNA should be extracted from fresh leaf material or material stored at -20C.
5. Using a mortar and pestle, finely grind the leaf material in the presence of liquid nitrogen
6. Transfer the ground plant material to a 50 ml Falcon tube
7. Add 8 ml of the activated CTAB buffer to the ground plant material
8. Vortex the mixture thoroughly and incubate at 65 °C for 30 minutes

9. Mix the contents thoroughly by inversion and incubate for another 30 minutes.
10. Add an equal volume (8 ml) of chloroform:isoamyl alcohol (24:1) and mix by inversion
11. Centrifuge the mixture at 6000 x*g* for 12 minutes.
12. Remove the supernatant and add it to a new 50 ml Falcon tube
13. Add 5µg of DNase free RNase and incubate the mixture at 37 °C for 30 minutes.
14. Add an equal amount of phenol:chloroform:isoamyl alcohol (25:24:1) and mix by vortexing
15. Centrifuge at 6000 x*g* for 12 minutes16. Remove the supernatant and add an equal volume of propan-2-ol, place at -20 °C for one hour.
17. Centrifuge at 10 000 x*g* for 30 minutes, discard the supernatant
18. Wash the DNA with two volumes of 70% ethanol.
19. Repeat the wash step
20. Resuspend the DNA in DNase/RNase-free deionized water or TE buffer (10 mM Tris, 1mM EDTA, pH 7.5).

DNA Extraction protocol 2 (small scale for SNP genotyping from silica dried *Greyia* leaf material)

Greyia leaf material was sampled in the field by collecting three leaves, preferably at leaf flush stage (1.5-2.5 cm diameter each), and placing them in airtight zip-lock bags containing silica gel beads, desiccant (2-5 mm) (catalogue# 1.07735, Sigma-Aldrich) for at least one week. (Note: To date, PCR-amplifiable gDNA has been isolated with this protocol from dried Greyia leaf material in silica desiccant or dried herbarium material that has been stored for up to four years). The silica-dried leaf material (6 leaf punches ~ 30 mg) was ground to a fine powder using a Geno/Grinder 2010 (Cole-Parmer). Genomic DNA was extracted using an oKtopure DNA extraction robot with the sbeadex Maxi Plant DNA Purification Kit (LGC Genomics GmbH) following the manufacturer's protocol with minor modifications.

Briefly, 250 µL Lysis Buffer PVP (LGC Genomics GmbH) was added to the ground tissue and samples were incubated overnight at 65 °C. After centrifugation at 2,500 × g for 10 min, 200 µL of the lysate was transferred to a new tube containing 520 µL Binding Buffer PN and 60 µL sbeadex magnetic particle suspension. The reaction solution was mixed thoroughly by pipetting, and incubated for 4 min at room temperature to allow DNA binding to the magnetic particles. Magnetic particles were captured on a magnetic stand and sequentially washed with 400 µL Wash Buffer PN1 and 400 µL Wash Buffer PN2. An optional brief wash step with 400 uL ultrapure water at room temperature can be included prior to elution. DNA was then eluted in 2 x 75 µL Elution Buffer PN after incubation at 55 °C for 10 min. The eluate was transferred to a new tube, evaporated to dryness, and resuspended in 50 µL elution buffer. Recovery of *Greyia* gDNA was ~ 50 ng/ µL on average. This protocol did not completely remove secondary metabolites and other potential PCR inhibitors, so dilutions of 1/10 to 1/100 were required for successful PCR amplification in some cases.

3RAD library preparation and sequencing

We prepared RADseq libraries using the Adapterama III library preparation protocol ((Bayona-Vasquez et al. 2019); their Supplemental File SI), that uses three restriction enzymes for digesting genomic DNA (3RAD), which is a modified version of double-digest (dd)RAD (Peterson et al. 2012).

For each sample, ~100 ng of genomic DNA were digested for 1 h at 37 °C in a solution with 1.5 µl of 10x Cutsmart® buffer, 0.25 µl (NEB®) of Read 1 enzyme (*XbaI, NEB*®) at 20 U/µl, 0.25 µl of Read 2 enzyme (*EcoRI-HF*, NEB®) at 20 U/µl, 0.25 µl of Read 1 adapter dimer-cutting enzyme (*NheI*) at 20 U/ µl, 1 µl of i5Tru adapter at 2.5 µM, 1 µl of i7Tru adapter at 2.5 µM and 0.75 µl of dH_2O.

After digestion/ligation, samples were pooled and cleaned with 1.2x Sera-Mag SpeedBeads (Fisher Scientiifc™) in a 1.2:1 (SpeedBeads:DNA) ratio, and the cleaned DNA was eluted in 60 µL of TLE.

An indexing PCR of the bead-purified sample pool was carried out with 10 µl of 5x Kapa Long Range Buffer (Kapa Biosystems, Inc.), 0.25 µl of KAPA LongRange DNA Polymerase at 5 U/µl, 1.5 µl of dNTPs mix (10 mM each dNTP), 3.5 µl of $MgCl_2$ at 25 mM, 2.5 µl of iTru5 primer at 5 µM, 2.5 µl of iTru7 primer at 5 µM and 5 µl of pooled DNA. The i5 and i7 adapters ligated to each sample using a unique combination (2 i5 X 1 i7 indexes).

The temperature conditions for PCR enrichment were 94 °C for 2 min of initial denaturation, followed by 10 cycles of 94 °C for 20 sec, 57 °C for 15 sec and 72° for 30 sec, and a final cycle of 72 °C for 5 min. The enriched samples were each cleaned with magnetic beads as described above, and quantified with a Quantus™ Fluorometer. Cleaned and quantified library pools per design were pooled to equimolar concentrations and were sent to the Norwegian Sequencing Centre (NSC) for quality control and subsequent final size selection using a one-sided bead clean-up (0.7:1 ratio) to capture 550 bp +/- 10% fragments, and the final paired-end (PE) 150 bp sequencing on one lane of the Illumina HiSeq 4000 platform.

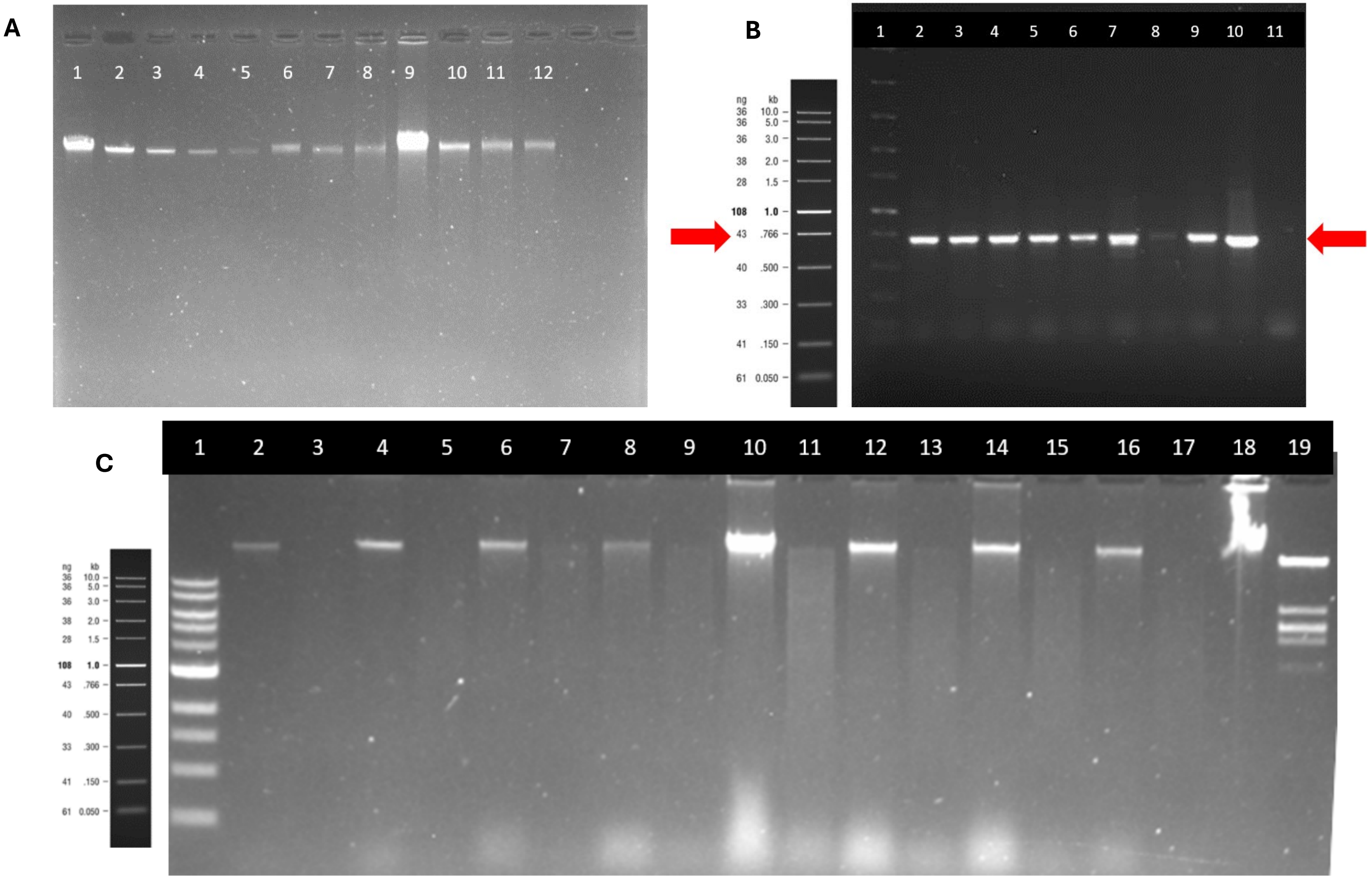

Figure S1. Quality checks performed on gDNA used for 3RAD library construction. (**A**) Qualitative and quantitative agarose gel indicating the integrity of the *Greyia* DNA. The concentrations of the DNA can be estimated when compared to known concentrations of the lambda DNA (supplier) dilution. The undigested lambda DNA was used as a size marker (48.5 kb) and 1ul of each sample was added to illustrate. In lane 1, 2, 3 and 4, lambda DNA was loaded at 67ng, 33ng, 16.5ng and 8ng respectively. *G. sutherlandii* (GSU) samples (P090_G60, P242_G64, P245_G65) were loaded in lanes 5, 9 and 10 respectively. *G. radlkoferi* (GRA) samples (P023_G61, P004_G66, P005_G67) were loaded in lanes 6, 11 and 12 respectively. *G. flanaganii* (GFL) samples (P160_G62, P161_G63) were loaded in lanes 7 and 8 respectively. **(B)** Continued on next page.

Fig. S1 – legend (continued)

(B) The *internal transcribed spacer (ITS)* region was PCR amplified from *Greyia* gDNA samples using ITS4 and ITS5 primers. DNA samples were diluted prior to the PCR reactions, since undiluted DNA contains PCR inhibitors. The dilution factors from stock concentrations are indicated in brackets. The NEB fast ladder (New England biolabs) was loaded in lane 1 followed by 3 µl of each PCR product. GSU samples P090_G60 (100ng/µl), P242_G64 (50ng/µl) and P245_G65 (150ng/µl) were loaded in lanes 2, 6 and 7 respectively. GRA samples P023_G61 (100ng/µl), P004_G66 (10ng/µl), P005_G67 (100ng/µl) were loaded in lanes 3, 8 and 9 respectively. GFL samples P160_G62 (100ng/µl), P161_G63 (100ng/µl) were loaded in lanes 4 and 5 respectively. Lane 10 contained a positive Maize control (M12) and the last lane contained the negative control. The red arrows indicate the amplified products at expected size (appx 700bp). **(C)** Restriction enzyme (EcoRI) digest of *Greyia* DNA**.** *Greyia* samples were loaded as follows: lane 1 NEB FAST DNA Ladder, lane 2 P090_G60u (26 ng), lane 3 P090_G60d, lane 4 P023_G61u (37.5 ng), lane 5 P023_G61d, lane 6 P160_G62u (34.5 ng), lane 7 P160_G62d, lane 8 P161_G63u (57.5 ng), lane 9 P161_G63d, lane 10 P242_G64u (337.5 ng), lane 11 P242_G64d, lane 12 P245_G65u (87.5 ng), lane 13 P245_G65d, lane 14 P004_G66u (96.5 ng), lane 15 P004_G66d, lane 16 P005_G67u (47 ng), lane 17 P005_G67d, lane 18 Lambda DNA uncut, lane 19 Lambda DNA after EcoRI digestion. (u) = undigested DNA; (d) = EcoRI digested DNA. The digestion reactions contained the same amount of DNA as the undigested reactions. The absolute amounts between samples differed, indicated by differing band intensities on the gel. In the odd number lanes, successful digestion of gDNA can be visualized as loss of the intact genomic DNA band and the appearance of lower-molecular-weight fragments evident as smears. The smears in some lanes are not readily evident due to the low concentrations of DNA loaded in that lane.

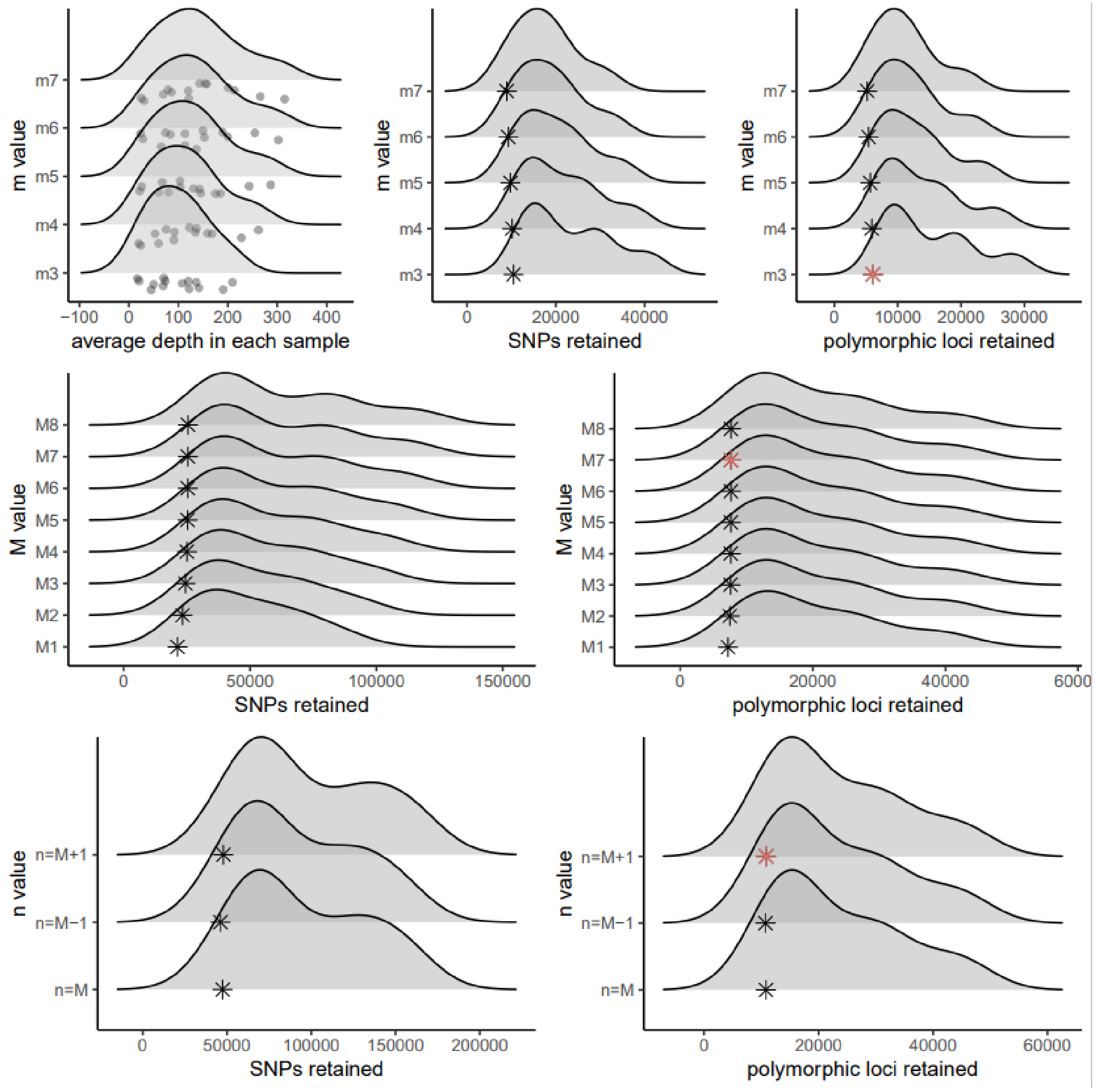

Figure S2. Parameter optimisation for SNP calling. Density plots showing the distribution of the number of 3RAD loci retained in the eight *Greyia* samples at different values of *m*, *M* and *n* values using the sequential STACKS parameter optimisation approach and *r80* loci rule. The optimal parameter sets that gave the highest number of polymorphic loci in 80% of the samples (*m* = 3, *M* = *7* and *n* = *8*) are indicated by red asterisks.

References

Bayona-Vasquez NJ, Glenn TC, Kieran TJ, Pierson TW, Hoffberg SL, Scott PA, Bentley KE, Finger JW, Louha S, Troendle N, Diaz-Jaimes P, Mauricio R, Faircloth BC (2019) Adapterama III: Quadruple-indexed, double/triple-enzyme RADseq libraries (2RAD/3RAD). PeerJ 7:e7724. doi:10.7717/peerj.7724

Botha I, De Canha MN, Oberlander K, Botes J, Lall N, Berger DK (2026) DNA barcoding and anti-tyrosinase activities of three species-representative populations of the genus Greyia Hook & Harv. South African Journal of Botany 189:55–67. doi:10.1016/j.sajb.2025.11.035

Peterson BK, Weber JN, Kay EH, Fisher HS, Hoekstra HE (2012) Double Digest RADseq: An Inexpensive Method for De Novo SNP Discovery and Genotyping in Model and Non-Model Species. PLoS ONE 7 (5):e37135. doi:10.1371/journal.pone.0037135
